# Quantitative Profiling of Single Formalin Fixed Tumour Sections: proteomics for translational research

**DOI:** 10.1038/srep34949

**Published:** 2016-10-07

**Authors:** Christopher S. Hughes, Melissa K. McConechy, Dawn R. Cochrane, Tayyebeh Nazeran, Anthony N. Karnezis, David G. Huntsman, Gregg B. Morin

**Affiliations:** 1 Canada’s Michael Smith Genome Sciences Centre, British Columbia Cancer Agency, Vancouver, British Columbia, Canada; 2 Department of Human Genetics, Research Institute of the McGill University Health Network, McGill University, Montreal, Canada; 3 Department of Molecular Oncology, British Columbia Cancer Agency, Vancouver, British Columbia, Canada; 4 Department of Pathology and Laboratory Medicine, University of British Columbia, British Columbia Cancer Agency, Vancouver, British Columbia, Canada; 5 Department of Medical Genetics, University of British Columbia, Vancouver, British Columbia, Canada

## Abstract

Although re-sequencing of gene panels and mRNA expression profiling are now firmly established in clinical laboratories, in-depth proteome analysis has remained a niche technology, better suited for studying model systems rather than challenging materials such as clinical trial samples. To address this limitation, we have developed a novel and optimized platform called SP3-Clinical Tissue Proteomics (SP3-CTP) for in-depth proteome profiling of practical quantities of tumour tissues, including formalin fixed and paraffin embedded (FFPE). Using single 10 μm scrolls of clinical tumour blocks, we performed in-depth quantitative analyses of individual sections from ovarian tumours covering the high-grade serous, clear cell, and endometrioid histotypes. This examination enabled the generation of a novel high-resolution proteome map of ovarian cancer histotypes from clinical tissues. Comparison of the obtained proteome data with large-scale genome and transcriptome analyses validated the observed proteome biology for previously validated hallmarks of this disease, and also identified novel protein features. A tissue microarray analysis validated cystathionine gamma-lyase (CTH) as a novel clear cell carcinoma feature with potential clinical relevance. In addition to providing a milestone in the understanding of ovarian cancer biology, these results show that in-depth proteomic analysis of clinically annotated FFPE materials can be effectively used as a biomarker discovery tool and perhaps ultimately as a diagnostic approach.

The rapid evolution of genome sequencing technologies has driven the growth of research for quantitative in-depth analysis of patient samples in a clinical setting. While mass spectrometry (MS)-based proteomics would provide highly informative data to support such research, proteome quantification in clinical materials has not been practical. The limited adoption of proteomics in these areas stems primarily from the poor performance of standard MS based approaches to work efficiently with practical quantities of formalin fixed paraffin embedded (FFPE) samples that are the standard media for clinical diagnostics^1^,^2^. Despite these challenges, recent research efforts have successfully obtained high quantitative coverage of the proteome from FFPE tissues using MS^3^,^4^,^5^,^6^,^7^. Problematically, these examinations have utilized quantities of tissues (e.g. whole resected tumours, milligrams of protein material, or more than ten 10 μm thickness scrolls of a block) that are not practical in clinical research samples that are often limited in addition to being chemically fixed.

In order to capture high quantitative coverage of the proteome from FFPE tumours on a more practical scale, recent studies have combined filter-based digestions (FASP) with MS1 quantification of peptide abundance (e.g. label-free)^8^,^9^,^10^. Using laser-capture microdissection (LCM) to enrich for cell populations of interest across multiple tissue sections, quantitative coverage of the proteome ranging from 5,000–10,000 unique proteins per sample was achieved from just 175–250 nL of cell volume (5–24 μg of peptides). Despite impressive results, these workflows have potential limitations related to the absolute recovery of protein material^8^,^11^,^12^, the number of individuals that can be practically examined due to sample processing and MS acquisition times (~24 hours for a single sample), and strict requirements for system configuration and stability due to the quantification method used (label-free). Therefore, extending the use of proteomics in clinical research and particularly in the clinical trials setting, which is the test bed for biomarker research, requires further development of robust and scalable technologies for FFPE tissue analysis by MS.

Elucidation of the functional processes that underlie the phenotype of a specific cancer can aid in the development of clinical treatment regimes. In ovarian carcinoma, histopathological examination has led to a clear definition of histotypes (e.g. high- and low-grade serous, endometrioid, clear cell, mucinous, and undifferentiated)^13^,^14^,^15^,^16^,^17^. However, clinical treatment protocols are only now being stratified by histotype. Large-scale screens have revealed diverse patterns of genome and transcriptome variation within and between histotypes^18^,^19^. Unfortunately, the extent to which the proteomes of these cancers differs is not known, as few studies have focused on in-depth differential quantitative analysis using MS technology^20^. Large-scale transcriptome analyses on their own often fail to correctly predict the complex network of protein changes that drive phenotype^1^,^21^, an inference that can be improved by investigating proteome variation through the use of MS technologies. Therefore, a high-sensitivity MS-based analysis of ovarian cancer histotypes can potentially build upon gene expression and immunohistochemistry (IHC) data to identify targetable proteins that drive different clinical phenotypes.

To demonstrate the ability of proteomics to provide robust insight into cancer biology from practical quantities of non-dissected clinical materials, we have developed a novel optimized pipeline termed SP3-CTP (SP3-Clinical Tissue Proteomics) and applied it to investigate the differential protein expression patterns that characterize ovarian high-grade serous (HGSC), clear cell (CCC), and endometrioid (ENOC) carcinoma histotypes. The SP3-CTP method enabled acquisition of the first in-depth quantitative proteome map that differentiates the main histotypes of ovarian carcinoma, revealing novel variation and protein features. Together, these data validate the ability of the SP3-CTP pipeline to generate actionable clinical information from practical amounts of FFPE tissue materials, and represents a significant advance in the understanding of histotype variation in ovarian cancer.

## Results

### Robust quantification of protein expression in FFPE tissues

**Figure 1:**
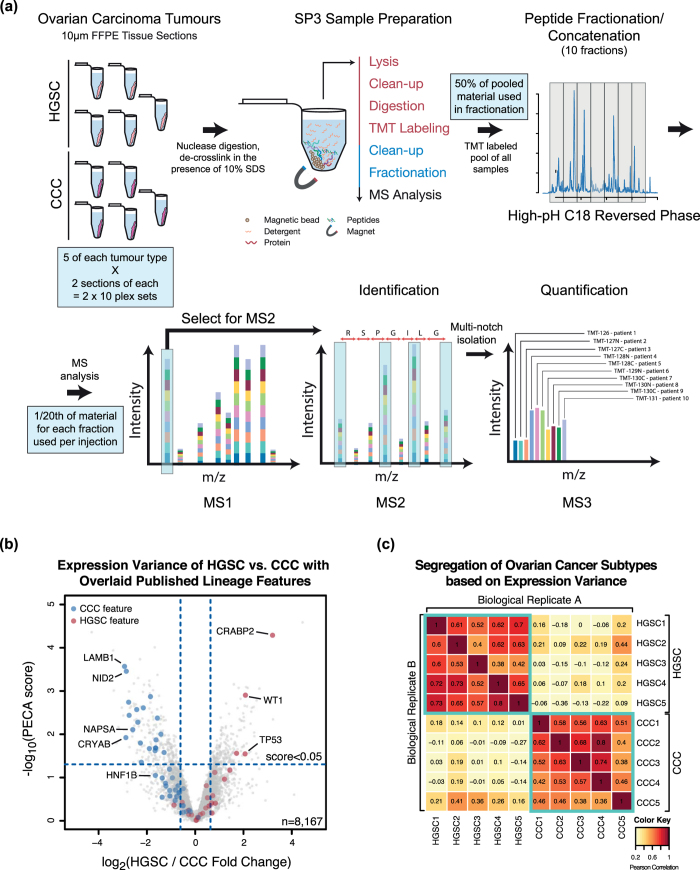
Robust proteomic analysis of FFPE tissue sections can be obtained with SP3-CTP. Sets of 10 unique tumour samples (5 HGSC, 5 CCC) were prepared in biological duplicate (serial sections) for proteomics analysis using SP3-CTP. Samples were analyzed in two separate 10-plex TMT experiments on an Orbitrap Fusion MS with MS3 scanning. **(a)** Schematic depicting the processing and analysis pipeline used with SP3-CTP. **(b)** Volcano plot depicting differential expression analysis between the two ovarian carcinoma histotypes (HGSC and CCC) using data from the combined analysis of Set A and Set B. Highlighted points are the those detected from the set of 113 genes previously identified as differentially expressed in analyses of HGSC and CCC tumour samples with RNA and antibody-based protein measurements. PECA score represents the median adjusted p-value of all peptides assigned to a protein. Dotted vertical lines indicate one standard deviation from the mean fold change. **(c)** Clustering heat-map depicting the reliable segregation of ovarian carcinoma histotypes based on protein expression patterns. The X-axis displays the clustering for the first biological replicate (Set A), and the Y-axis for Set B (second biological replicate). The top 1000 peptides that contributed to differential expression between the HGSC and CCC histotypes were used in this comparison (n = 261 unique proteins for biological replicate A, n = 271 unique proteins for biological replicate B).

To build a platform that yields reproducible and robust results from clinical FFPE tissues, we designed a workflow based on the previously established paramagnetic bead method, SP3^22^. SP3 uses hydrophilic interaction where biomolecules are trapped in a solvation layer on the surface of the beads in the presence of organic solvent, permitting the manipulation of protein and peptide mixtures in an unbiased manner. SP3 was recently shown to provide enhanced performance in the analysis of quantity-limited cell samples when compared with high efficiency filtration and stage tip methods^22^. For the analysis of FFPE tissue sections we modified the method to a simple and practical single-tube, solution-based procedure for nuclease pre-digestion, lysis and de-crosslinking in the presence of >10% SDS, pre-digest clean-up, proteolysis, and tandem mass tag (TMT) labeling^23^. High proteome coverage and accuracy in quantification was enabled with high-pH C18 reversed phase fractionation and MS3 analysis on an Orbitrap Fusion (Fig. 1a).

To determine the performance of SP3-CTP for the study of clinical cancer materials, we examined an initial set of FFPE tumour tissue samples derived from a collection of HGSC or CCC ovarian carcinomas (Supplemental Table 1). Two TMT 10-plex sets were constructed (Set A and Set B) with one 10 μm scroll per tube from each of 5 HGSC and 5 CCC FFPE tumour blocks (20 total scrolls), and analyzed using the SP3-CTP workflow (Fig. 1a). A 10 μm thickness represented a balance between sufficient tissue for proteome analysis and a readily available amount of clinical material. Based on analysis of representative tissue sections (n = 3), a single, non-dissected 10 μm scroll (~1 cm × ~1.5 cm average) yields ~82 ± 15.0 μg of protein (BCA assay), translating to ~72 ± 18.2 μg of peptide (UV spectrophotometry) with SP3-CTP (88% recovery). These values are in agreement with previous assays of protein yield from un-dissected tissue sections^24^, and illustrate the high efficiency of the SP3-CTP approach.

The MS analysis of the combined data from both proteome sets (Set A and Set B) yielded a total of 8,167 proteins quantified, with 7,854 of these having a matched RNA-seq read for the 12,974 transcripts (~51%) expressed with fragments per kilobase of transcript per million mapped reads (FPKM) value of 1 or greater in normal ovarian tissue from one individual in the Human Protein Atlas (HPA)^25^ (Supplemental Fig. 1a). From the set of RNAs with an FPKM value greater than 10 which were not identified in the SP3-CTP data, there was enrichment in proteins with transcription factor activity and nuclear localization (FDR adjusted p-values = 7.3e-06, 6.9e-06) as annotated by Gene Ontology (GO). Examining the peptide prevalence in the identification matrix revealed that there was a very high sampling density between tumours, with 98.5% of the 8,167 proteins quantified in all 10 individuals with no missing peptide values (Supplemental Fig. 1b,c). We observed excellent correlation between both technical (repeat injections of one set (e.g. Set A1 vs. A2, Set B1 vs. B2); mean r^2^ = 0.84–0.86) and biological replicates (serial sections; Set A vs. Set B; mean r^2^ = 0.75–0.76) (Supplemental Fig. 2a,b). The analysis of an added *E. coli* lysate standard for intra-batch normalization revealed only small variations in processing, indicating that the observed expression diversity stems from true biological differences between tumours or histotypes, rather than the SP3-CTP protocol itself (Supplemental Fig. 2c,d).

Comparing HGSC with CCC highlighted the enriched expression of specific proteins previously described for ovarian carcinoma, such as: HNF-1β, NID2, NAPSA, and CRYAB for CCC, and CRABP2, TP53, and WT1 for HGSC (Fig. 1b)^26^. Interestingly, numerous CCC protein features, such as HNF-1β and NAPSA, had FPKM values of 1 or below in the HPA RNA-seq analysis of a normal ovarian tissue sample^25^. The transcripts with FPKM <1 that were identified in the proteomics data were enriched for proteins with a plasma membrane, extracellular region, and cytoskeleton (FDR adjusted p-values = 4.9e-03, 2.2e-14, 5.3e-04) GO annotation. In addition, a large portion of these lowly expressed RNAs were annotated as single peptide identified proteins in the SP3-CTP data (56% of 937 proteins). As is typical in MS analyses, ~20% of the proteins represented in the overall data originate from single peptide hits (Supplemental Fig. 3a), however the majority of significant differentially expressed candidates stem from multi-peptide identified proteins (Supplemental Fig. 3b). Using proteins identified from a set of 113 genes generated from HGSC and CCC signatures determined to be histotype-specific based on mRNA microarray or IHC analyses^18^,^27^,^28^,^29^, we observed clear patterns of differential expression between the histotypes (Fig. 1b, Supplemental Table 2). Using the top 1000 differentially expressed peptides in either the HGSC or CCC individuals as feature sets (n = 261 unique proteins for Set A, n = 271 unique proteins for Set B), clear and reproducible tumour histotype clustering was achieved (Fig. 1c).

### Clinical tissues accurately reflect the biology of ovarian cancer

**Figure 2:**
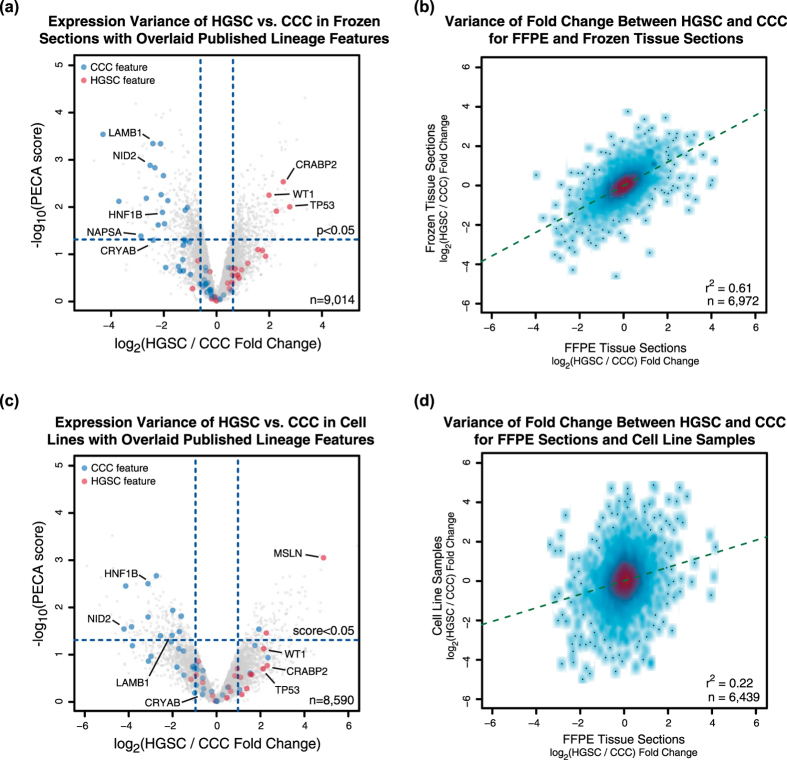
Biological variance derived from FFPE samples representing ovarian carcinoma histotypes can be recapitulated in samples from different source materials. Sets of 10 unique FFPE tumour samples (5 HGSC, 5 CCC) were prepared in biological duplicate using SP3-CTP. Alongside, a set of 8 (4 HGSC, 4 CCC) matched frozen tissue sections, and 6 cell lines (3 HGSC, 3 CCC) were prepared in biological duplicate and analyzed using the same pipeline. (**a**) Volcano plot depicting the variance in expression between the HGSC and CCC histotypes in the frozen tissue section data. Highlighted points indicate proteins identified from the 113-gene feature set previously shown to differentiate these ovarian carcinoma types. The dotted vertical lines indicate one standard deviation from the mean fold change. (**b**) Density smoothed scatter depicting the correlation of fold change values obtained from the FFPE and frozen tissue section data when comparing HGSC with CCC. (**c**) Volcano plot depicting the variance in expression between the HGSC and CCC histotypes in the cell line data. Highlighted points indicate proteins identified from the 113-gene feature set previously shown to diverge between these ovarian cancer types. The dotted vertical lines indicate one standard deviation from the mean fold change. (**d**) Density smoothed scatter depicting the correlation of fold change values obtained from the FFPE and cell line data when comparing HGSC with CCC. All correlation values were calculated using the Pearson method.

Although the analysis of 5 HGSC and 5 CCC tumours displayed established patterns of protein expression characteristic of ovarian cancer histotypes, it remained unclear whether the FFPE treatment of the material inhibited accurate in-depth quantitative proteome analysis with SP3-CTP. To determine if clinical FFPE tissues can yield proteome values that accurately reflect established patterns in ovarian cancer pathology, we compared the FFPE samples with matched frozen tumour materials (Supplemental Table 1). We analyzed two serial frozen sections from each of 8 individuals (4 HGSC, 4 CCC; two TMT 8-plex sets, one section per tumour in each) where matched material was available. The analysis quantified 9,014 proteins, with 7,624 proteins (~59% of 12,974 transcripts) having a matched RNA-seq read with FPKM ≥1 in the ovarian tissue analysis (Supplemental Fig. 4a). Similar to the FFPE samples, comparing HGSC to CCC using the biological replicate frozen tissue samples yielded fold-change values that were highly reproducible (Supplemental Fig. 4b), and recapitulated the expected variance for proteins from the 113-gene set (Fig. 2a). Although frozen sections appeared to yield enhanced sensitivity compared to FFPE when considering depth of coverage (9,014 vs. 8,167 proteins), the observed trends in expression variance between HGSC and CCC were highly correlated (Fig. 2b).

To compare differential expression with *in vitro* cultured materials that represent the desired ovarian carcinoma histotypes, 3 HGSC (OVCAR-3, OVCAR-5, OVSAHO) and 3 CCC (JHOC-5, OVISE, OVTOKO) cell lines (Supplemental Table 1) were analyzed using SP3-CTP (Supplemental Fig. 5a). The analysis quantified 8,590 proteins, with 7,457 (~57% of 12,974 transcripts) having a matched RNA-seq read with FPKM ≥1 in the HPA ovarian dataset (Supplemental Fig. 5b). Similar to the FFPE and frozen samples, the cell line data exhibited high reproducibility for differential expression between biological replicates for the HGSC to CCC comparison (Supplemental Fig. 5c), and the expected variation in proteins from the 113-gene set (Fig. 2c). However, the expression values correlated more strongly for the FFPE and frozen material (r^2^ = 0.61 for peptides) than with the cell lines (r^2^ = 0.22, Fig. 2d), indicating the potential loss of histotype fidelity when using cultured materials. Altogether, these data highlight the ability of clinical FFPE tissue materials to yield protein expression patterns representative of ovarian cancer biology that are poorly manifested by analysis of cultured cell lines.

### Mapping the histotype-specific proteomes of ovarian carcinoma

To carry out an in-depth examination of the three major ovarian carcinoma histotypes (HGSC, CCC, and ENOC) using the validated SP3-CTP workflow, we selected a set of 18 individuals (6 of each histotype) and scrolled serial 10 μm FFPE sections in triplicate for each individual (54 total sections) (Supplemental Fig. 6a). These data resulted in 9,049 quantified proteins, with 7,575 proteins (~58% of 12,974 transcripts) having a matched RNA-seq read in the ovarian HPA (FPKM ≥ 1) dataset (Supplemental Fig. 6b). Importantly, the normalized log2 expression values for the *E. coli* peptides were found to be highly reproducible across samples and batches (Supplemental Fig. 6c), indicating the stability of the processing and analysis protocols across all sets.

**Figure 3:**
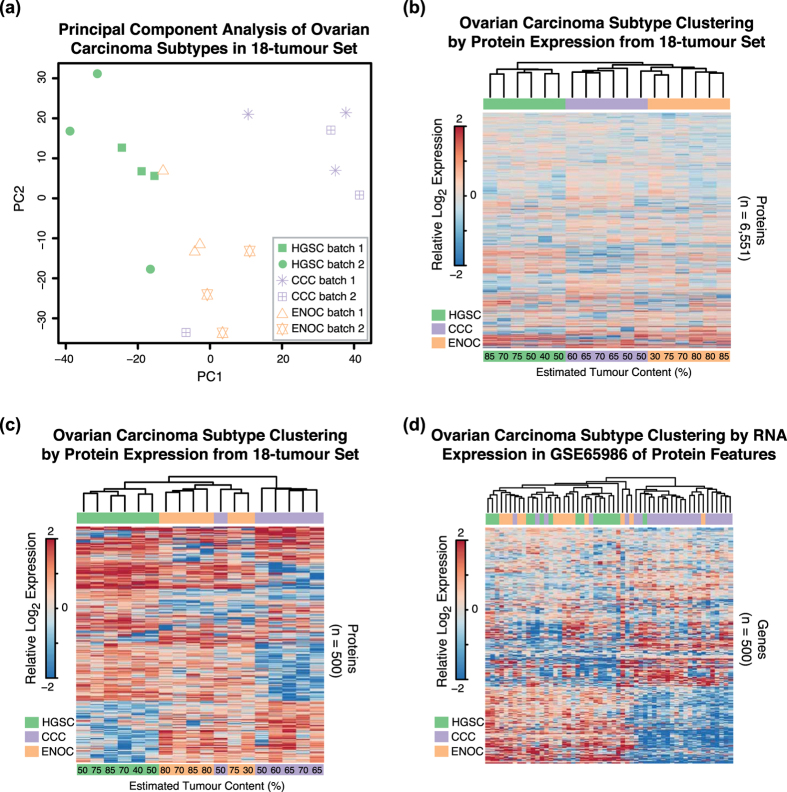
Ovarian carcinoma histotypes can be robustly segregated using proteome expression maps generated using SP3-CTP. From the analysis of the set of 18-tumour samples covering HGSC, CCC, and ENOC, histotypes were cross-compared to clusters of gene expression patterns. In all analyses, relative log2 expression values that represent the difference of abundance in a single individual relative to the median across all individual tumours per gene were used. **(a)** PCA of the 18-tumour proteomics data performed using the prcomp function in R. Samples examined in different batches are denoted by different symbols, and histotypes by matching colors. **(b)** Heat-map depicting unsupervised hierarchical clustering of the 18-tumour samples using the total set of proteins identified with an expression value across all tumours. Tumour content values are estimated from histological analysis. **(c)** Heap-map depicting unsupervised hierarchical clustering of the 18-tumour samples using a subset of the top 500 proteins that have the largest contribution to variance as determined from principal component analysis. **(d)** Heat-map depicting unsupervised hierarchical clustering of the 55 tumour samples analyzed for RNA expression in GSE65986^30^. The 500 genes used in clustering are derived from the proteomics data from PCA as in **(c)**.

To obtain an initial estimate of how well the proteomics data segregated ovarian cancer histotypes we performed unsupervised clustering of the entire cohort across all batches. Principal component analysis (PCA) revealed that the individuals with matching histotypes clustered largely together (Fig. 3a), indicating that distinct patterns of protein expression were present within each histotype set. Importantly, the PCA arranged individuals independent of batch, demonstrating that the observed groupings were driven by biological variation between HGSC, CCC, and ENOC rather than from technical artifacts in the MS analysis. Unsupervised hierarchical clustering using the set of proteins identified in all tumours with no missing values (n = 6,551) correctly grouped the six tumours of each histotype (Fig. 3b). Using only the top 500 proteins that contributed to the variance in the PCA revealed differential expression patterns between the histotypes (Fig. 3c). With the same set of 500 proteins, we attempted to cluster the histotypes using their mRNA expression values from a microarray data analysis of 55 ovarian tumours^30^ (Fig. 3d). Only the CCC samples exhibited a strong differential expression pattern, with the HGSC and ENOC samples forming an intermixed cluster in the RNA data.

To functionally classify proteins observed to be highly expressed relative to the other histotypes in the proteomics data, we performed a GO analysis using Metascape^31^ (Supplemental Table 3). Relative to CCC and ENOC, significant enrichment in HGSC of proteins involved in small molecule metabolism (log10 q-value = −7.3), immune response (log10 q-value = −3.8), and response to DNA damage (log10 q-value = −3.0) were observed. In CCC, enrichment of proteins involved in endocytosis (log10 q-value = −10.1), inflammatory response (log10 q-value = −9.3), and wound healing (log10 q-value = −10.1). In ENOC, enrichment of proteins involved in organization of the extracellular matrix (log10 q-value = −7.0), adhesion (log10 q-value = −6.3), and actin filament polymerization (log10 q-value = −7.3) were identified.

To further resolve the above ontologies at the level of functional gene sets, proteins from each histotype were mapped to the Molecular Signature Database^32^ Hallmark collection with Metascape (Supplemental Table 3) using gene set enrichment analysis (GSEA) (Supplemental Table 4). For proteins with high expression in HGSC, significant enrichment of interferon response (log10 q-value = −19.2), fatty acid metabolism (log10 q-value = −7.3), and adipogenesis (log10 q-value = −7.4) were observed. Global GSEA corroborated the interferon response signature in HGSC (p-value = 2.0e-08). Analysis of CCC revealed enrichment of proteins involved in xenobiotic metabolism (log10 q-value = −9.5) and adipogenesis (log10 q-value = −9.5). GSEA further corroborated the elevated expression of proteins involved in xenobiotic metabolism (p-value = 0.014) in the CCC histotype. The ENOC histotype data revealed an inverse enrichment of interferon response (p-value = 1.1e-05) when compared to HGSC in GSEA analysis.

To investigate the profiles of proteins characteristic for biological processes found in specific ovarian carcinoma histotypes, we examined a gene signature (322 unique genes in the signature set) for microenvironmental pathology in CCC taken from a global microarray mRNA expression analysis of 38 cell lines^33^. This signature contains known markers of CCC, such as HNF-1β, and others consistent with oxidative stress. Evaluation of this signature revealed that only 26 of the genes mapped to the set of top 500 proteins contributing to the variance between HGSC and CCC in the SP3-CTP data (183/322 of the signature genes are mapped in the total set of SP3-CTP data). However, overlaying the gene signature with our proteomics data revealed the correlated expression of the vast majority of signature genes (Supplemental Fig. 7a). Furthermore, a subset of the top 75 genes (75/183 identified) from the signature set that contributed the most to the variance between subtypes in the SP3-CTP data facilitated robust segregation of CCC cases from those classified with HGSC or ENOC (Supplemental Fig. 7b,c). This demonstrates that this characteristic gene subset displays attributes that span the transcriptome and proteome, and is indicative of the CCC histotype when compared with HGSC and ENOC.

### Developing protein feature profiles of ovarian carcinoma using SP3-CTP MS data

To evaluate the accuracy of the quantitative variance observed between the defined histotypes in the SP3-CTP data, we compared it with microarray-derived mRNA expression values from an analysis of 55 ovarian cancer tumour samples^30^. Focusing on the 6 HGSC and 6 CCC tumours revealed significant differences in protein abundance between these histotypes that is also reflected at the RNA level, and specifically for features in the 113-gene set (Supplemental Fig. 8a). The trends observed for differential expression between the protein and RNA data for HGSC and CCC mirrored those from the previous FFPE and frozen sets (Supplemental Fig. 8b,c), whereas the cell line data exhibited increased deviation (Supplemental Fig. 8d). The differential expression patterns observed when comparing the HGSC and CCC histotypes with ENOC (Supplemental Fig. 9a,b) exhibited lower correlation with RNA variance than those between the other histotypes (Supplemental Fig. 9c,d).

**Figure 4:**
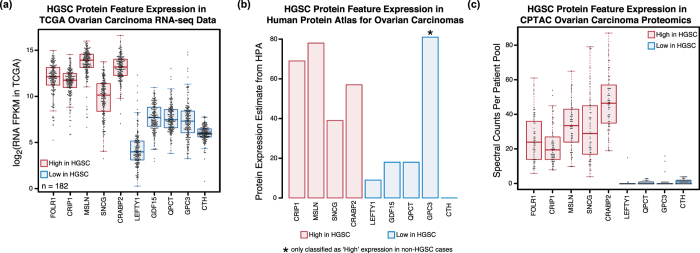
Integrated interrogation of large-scale transcriptome and proteome data using SP3-CTP derived candidates highlights the differential biology of ovarian carcinoma histotypes. From the analysis of a set of 18-tumour samples from unique individuals covering HGSC, CCC, and ENOC, histotypes were cross-compared to generate sets of enriched markers for each. (**a**) Boxplots depicting the RNA expression in the TCGA panel of HGSC ovarian cancer tumours of 5 proteins (FOLR1, CRIP1, MSLN, SNCG, CRABP2) found to have high, and 5 (LEFTY1, GDF15, QPCT, GPC3, CTH) with low expression in HGSC determined in the SP3-CTP proteomics data. Overlaid points indicate the expression of these proteins in each individual tumour of that type. The total number of tumours in the TCGA dataset used was 182. (**b**) Expression of the 10 SP3-CTP-derived ovarian cancer proteins in the IHC data taken from the HPA^34^. The HPA data (n = 12 total individuals) contains values for multiple ovarian carcinoma histotypes that are aggregated for this analysis. Expression values were calculated based on assigning a numerical score of 9 for ‘High’, 6 for ‘Medium’, 3 for ‘Low’, and 0 for ‘Not detected’. Each expression value was multiplied by the number of tumours assigned with that class, and the total sum calculated to generate a per protein expression estimate. (**c**) Expression of the 5 candidate HGSC proteins in MS-based proteomics data taken from the CPTAC study of ovarian cancer. Values are spectral counts calculated across all pools of samples taken from studies completed at the two CPTAC involved institutes (Supplemental Table 5).

To determine whether the observed expression trends could be validated in cohorts beyond the set analyzed by SP3-CTP, we overlaid our results with those from the transcriptome analysis of ovarian HGSC carried out by The Cancer Genome Atlas (TCGA)^19^. We extracted a set of 10 proteins (5 higher, 5 lower in HGSC) from the MS data that exhibited reliable differential expression and have either established roles or are novel in the context of ovarian or other cancers (Supplemental Fig. 10). Examining TCGA FPKM values derived from RNA-seq of 182 ovarian tumours (primarily HGSC), we observed the expected expression trends for the HGSC features derived from the proteomics data (Fig. 4a). Taking the protein candidates with the highest (MSLN) and lowest (LEFTY1) expression in HGSC relative to the other histotypes and mapping these across other cancers (Supplemental Table 5) revealed highly variable patterns of abundance between TCGA tissue type sets (Supplemental Fig. 11a,b). Surprisingly, only LEFTY1 was found to have significant promoter methylation among the five low expressed proteomics derived genes (Supplemental Fig. 11c). None of the 10 MS-derived proteins were found in a modeled set of genes predicted by the TCGA to be indicators of patient prognosis and the overall TCGA prognostic signature did not correlate with the HGSC histotype in the proteomic data (Supplemental Fig. 11d).

To corroborate the observed RNA expression trends from the TCGA data for the 5 high and 5 low proteomic HSGC features, we mapped the expression dynamics for these proteins using the cancer IHC data available from the HPA^34^. Although the HPA data contains values for non-HGSC ovarian tumours, they confirmed the expression patterns in ovarian carcinomas at the protein level for the majority of candidates (Fig. 4b, Supplemental Fig. 12a,b). We also queried the expression of HGSC features in a recent in-depth proteomic analysis of ovarian cancer (primarily HGSC)^5^ matched with the TCGA ovarian tumour cohort. Using spectral counts from the published data as a measure of expression, we observed similar trends across HGSC for the selected high and low proteins (Fig. 4c). This trend was not reflected in the isobaric tag-based abundance values (Supplemental Fig. 13a,b), likely due to the fact that these per-individual values are calculated relative to a study-specific pooled reference standard that make direct comparisons with the SP3-CTP data challenging.

**Figure 5:**
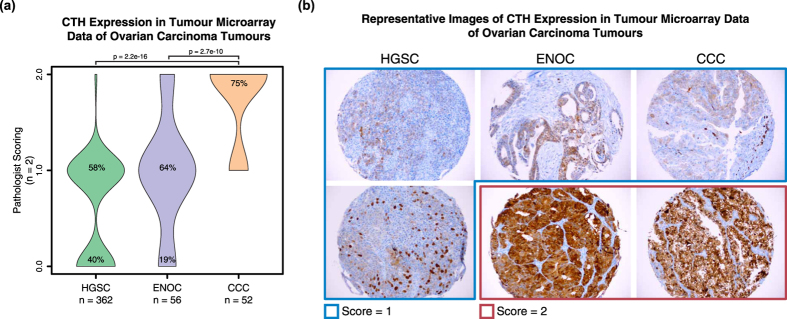
Validating ovarian cancer markers captured in MS-based proteomics screening on arrays of histotype-specific tumour materials. A TMA containing 485 ovarian cancers was stained for CTH. Shown are the data for the HGSC, CCC and ENOC histotypes. (**a**) Violin plot depicting the densities of individuals scored based on expression of the marker CTH. Histotypes are listed along the x-axis. Proportions of positively staining tumours is displayed as a percentage at the matched score level. Sections on the TMA were scored manually based on a system of 0 = negative, 1 = weak or variable, 2 = strong straining. P-values were calculated using a Mann-Whitney U-test. (**b**) Representative images showing the staining for each of the histotypes are shown.

In addition to known features and biomarkers of the histotypes, several novel candidate proteins emerged from the SP3-CTP data which had higher expression in specific individuals (supplemental data objects in Supplemental Table 6). We evaluated the abundance of cystathionine gamma-lyase (CTH) and LEFTY1 that were both enriched in CCC compared to HGSC and ENOC in the proteomics data (Supplemental Fig. 10) and across all public repositories (Fig. 4a–c). In a western blot analysis of a panel of ovarian cancer cell lines of validated histotype; the majority of CCC cell lines showed high expression of CTH and LEFTY1 (Supplemental Fig. 14a,b). Furthermore, in an IHC analysis of a TMA of 485 ovarian cancer samples 75% of CCC cases stained intensely for CTH in contrast to ENOC (17%) and HGSC (2%) (Fig. 5a,b). Taken together, these data demonstrate the orthogonal nature of the proteome data with established genome and transcriptome screens, and highlight the ability of SP3-CTP to yield actionable protein features with potential clinical relevance in the context of ovarian carcinoma.

## Discussion

In this work we have presented an improvement and novel application of the SP3 proteomics methodology that enabled high quality quantitative analysis of clinical FFPE tumour sections. SP3-CTP was leveraged to generate the first in-depth quantitative proteome analysis of ovarian carcinoma histotype variation from clinical FFPE tissue samples. Currently these data represent the largest quantitative analysis of the major ovarian carcinoma histotypes using MS-based proteomics techniques, and complements the large body of existing gene expression data used to derive candidate markers of these individual diseases. The derived proteome maps reveal characteristic patterns of protein expression that are histotype-specific, including established features of these individual diseases, and numerous novel candidates. When evaluated in comparison with the large bodies of gene expression data for ovarian carcinoma, robust extraction of reliable protein features with potential clinical relevance, such as CTH, is achieved.

Although SP3-CTP integrates a number of established proteomics tools, the simplicity of the optimized approach creates a robust single-tube pipeline for proteome analysis of practical amounts of FFPE tissues. SP3-CTP affords improvements in throughput and processing time through the use of magnetic beads and offers greater flexibility in sample handling by enabling the use of a wide range of reagents (e.g. high concentrations of detergents or chaotropic agents) that may be incompatible with conventional spin-filter units. Importantly, these advantages do not come at the cost of data quality, as SP3-CTP consistently achieved equivalent levels of quantitative proteome coverage when compared with currently established state-of-the-art workflows^4^,^5^,^6^,^7^,^10^. Although capture of material from FFPE tumour blocks can be affected by a number of factors (e.g. block age, fixation protocol, cellularity), SP3-CTP consistently demonstrated high-efficiency in protein and peptide recovery from single non-dissected FFPE tissues.

Integrated analysis of RNA and protein expression patterns is a valuable tool for revealing and confirming additional layers of regulatory variation between pathological conditions^35^. The correlation in global expression between SP3-CTP derived protein and RNA in the ovarian carcinoma histotype data was modest, with the highest agreement in the HGSC vs. CCC comparison. Numerous prior analyses have noted the discordance between protein and RNA levels^36^,^37^, highlighting the variance observed when making comparisons of high-coverage transcriptome and proteome datasets. However, recent studies utilizing individual-matched data have illustrated that a majority of transcript and protein pairs have positively correlated expression^3^. We also found that patterns in proteins identified in a 113-gene set with differential expression between HGSC and CCC correlated highly between the two types of data, with very few proteins showing opposite directionality for SP3-CTP and RNA expression. This trend carried across all material types, including that derived from cell lines, indicating the accuracy of the relative estimates of protein expression obtained with SP3-CTP when compared with orthogonal techniques. Notably, robust expression estimates of these known features were captured despite working with FFPE tissues that display heterogeneity in tumour content and variable sample acquisition age (years – 2008–2012).

Globally, the dynamics of the SP3-CTP protein sets reveals large groups of proteins that display histotype-specific expression patterns and that have been characterized in the context of ovarian and other cancers. For example, the folate receptor (FOLR1) is associated with different types of epithelial and ovarian carcinomas^38^ due to its specific appearance in these diseases. In the proteomics data, FOLR1 displays characteristic high expression in HGSC, with low levels found in CCC and ENOC. Recent work has demonstrated the association of FOLR1 expression and increased survival in the first 2 years following diagnosis with HGSC ovarian cancer^39^ and the inverse in CCC^39^. Members of the KLHL (Kelch-like) gene family have characterized associations with a variety of cancers^40^. In our results we found that KLHL14 was enriched in HGSC, however there are currently no studies characterizing the function of this gene product in ovarian cancer. Similarly, the BSN (bassoon presynaptic cytomatrix) protein displays histotype-specific expression in CCC and ENOC. BSN is linked with various neurological conditions^41^ but remains uncharacterized in the context of ovarian cancer.

Comparison with TCGA RNA expression, CPTAC protein, and HPA IHC datasets revealed the conserved histotype expression of additional features extracted from the SP3-CTP proteomic data. The cell surface glycoprotein encoded by the MSLN gene was found to have consistently elevated abundance at the transcript and protein levels in HGSC, a trend known to correlate with the CA125 antigen in ovarian cancer^42^. MSLN has been reported to have value as a prognostic marker, with highly enriched expression in HGSC ovarian cancer^43^. Conversely, LEFTY1 was observed to have consistently low expression in HGSC. LEFTY1 has established roles as a cell fate determinant in embryonic stem cells^44^ and as a negative regulator of the TGF-β superfamily member NODAL^45^. NODAL is specifically described to be involved with development of a cancer phenotype in a variety of cellular systems^46^,^47^,^48^. The presence of the LEFTY proteins in a human embryonic stem cell conditioned matrix^49^ is implicated in the reprogramming of metastatic melanoma cells through modulation of NODAL^50^. Despite the potential tumour-suppressor characteristics of LEFTY1 and the specific abundance profile of this protein, there is currently limited knowledge linking its expression to the pathology of ovarian cancer histotypes.

Of the HGSC-low expressed protein features relative to ENOC and CCC, of particular interest is the metabolic enzyme CTH. CTH is involved in the production of the cysteine precursor, cystathionine^51^. The production of cysteine is important for the generation of the antioxidant, glutathione. In ovarian cancer cell lines, depletion of the enzyme that catalyzes the metabolic step prior to CTH, cystathionine beta synthase (CBS), induces reactive oxygen species (ROS) accumulation leading to reduced mitochondrial respiration and ATP synthesis^52^. CBS was also observed by SP3-CTP to have significantly elevated expression in CCC relative to the other histotypes. Increased oxidative stress due to ROS in endometriotic cysts where some CCCs are thought to originate has been suggested to contribute to tumourigenesis^53^.

An established hallmark of CCC that is observed in the SP3-CTP data is increased hepatocyte nuclear factor 1β (HNF-1β) expression^53^,^54^,^55^. Increased levels of HNF-1β are suggested to play a role in the development of the ‘Warburg effect’ in CCC, conferring the advantage of enhanced cell survival through reduction of oxidative phosphorylation, and thereby exposure to ROS^53^. By maintaining precursors for glutathione synthesis, CTH may operate synergistically with HNF-1β to preserve an environment low in ROS to enhance survival. The consistently high expression of CTH and HNF-1β across a range of CCC data sets acquired using multiple acquisition techniques suggests that CTH may be a reliable marker for the CCC histotype. While we have observed high CTH expression in CCC carcinomas in a large TMA cohort our observations will need to be validated in large independent cohorts of ovarian carcinomas.

Overall, we have presented a novel high-resolution proteomic analysis of ovarian carcinoma covering the HGSC, CCC, and ENOC histotypes using the newly developed SP3-CTP method for analysis of clinical FFPE tumour material. The demonstrated reproducibility and robustness of the presented research platform highlights the enormous potential and practicality of the method to identify novel markers to aid in diagnosis, and identify protein targets for therapeutics in all cancer types through MS-based analysis of clinical materials.

## Materials and Methods

### Study Design

A total of 4 sample sets were utilized to enable accurate estimation of the protocol efficiency, as well as to develop reliable protein maps of the ovarian cancer histotypes. In the initial optimization analysis a set of 10 μm tissue sections derived from a total of 10 unique ovarian carcinoma tumours were used. For each tumour, two serial sections for each tumour block were taken to represent biological replicates. Each of these biological replicate sets was injected twice into the MS to represent technical duplicates (2 biological replicates × 2 injections × 10 tumours). In the comparison with the frozen tissue material, a total of 8 tumours with matched material from the initial FFPE screen were utilized. As with the FFPE, serial 10 μm tissue sections were treated as biological replicates, and multiple injections as technical (2 biological replicates × 2 injections × 8 tumours). In the comparison with cell-line samples, a total of 6 different lines were used. Individual cell pellets were treated as biological replicates, and multiple injections as technical replicates (2 biological replicates × 2 injections × 6 cell lines). In the final sample set, a total of 18 individuals were compared. Serial sections were treated as biological replicates and only a single injection was used for each (3 biological replicates × 1 injection × 18 individuals). In this set, a pooled standard was created by mixing an aliquot from all samples that was then used as the 10^th^ channel in each TMT 10-plex batch. This resulted in two sets of 10-plex samples (9 individuals + 1 standard) with 3 biological replicates for each. In the FFPE and 18-tumour set, each sample channel was spiked with a small amount of *E. coli* protein lysate prior to SP3 treatment to monitor batch effects and reproducibility. The concatenated peptide fractions for this final sample set were run in a randomized order on the MS to eliminate batch effects over the extended analysis time required.

### Tissue Sample Acquisition and Preparation

All tissues were obtained after informed consent of the patients under the supervision of the University of British Columbia, British Columbia Cancer Agency Research Ethics Board. All methods and experimental protocols were approved and carried out in accordance with guidelines established by the University of British Columbia, British Columbia Cancer Agency Research Ethics Board. Archival FFPE clinical ovarian cancer specimens were evaluated by a pathologist (T.N. or A.N.K.) to determine their histotype and cellularity (Supplemental Table 1). The average dimensions of the tumours sectioned in this work were ~1 cm × ~1.5 cm, with cellularity measurements ranging from 30–85% and tumour content values from 50–90%. In this work, cellularity refers to the area of the block that is covered by tumour tissue relative to surrounding normal. Tumour content denotes what proportion of this cellularity area is comprised of malignant cells relative to inflammatory and fibroblastic cells. To each 10 μm scrolled section, 1 mL of xylene (Sigma) was added, and vortexed for 10 seconds. Sections were centrifuged for 3 minutes at 15,000 g, and the xylene-containing supernatant removed and discarded. To each section, 1 mL of 100% ethanol was added, vortexed, centrifuged, and discarded. Sections were air dried for 10 minutes in a fumehood, and stored at −20 °C until use.

### SP3-CTP Tissue Lysis, Protein Reduction, and Alkylation

All tissue sections used (including frozen and FFPE) were individually lysed using a combination of enzymatic dissociation and heating. To each section, 30 μL of nuclease buffer consisting of 1% SDS, 100 Units of Benzonase (EMD Millipore), and 200 mM HEPES pH 8 was added and incubated at 37 °C for 1 hour. After nuclease treatment, 30 μL of 20% SDS was added, and the sample was heated for 45 minutes at 95 °C. Reduction and alkylation was performed through addition of 10 mM TCEP and 40 mM chloroacetamide (final concentrations) with incubation for 30 minutes at 37 °C. Representative tissue sections were measured for protein content using a BCA assay (Thermo Fisher). Samples were stored at −20 °C until SP3 treatment.

### SP3 Processing of Protein Samples

All protein samples processed (cell line and tissue) were handled using the below described SP3 protocol unless otherwise noted. To each protein mixture to be treated, 5 μL of each of the two types of beads used in SP3 (Supplemental Protocols) was added at this stage and mixed to generate a homogeneous solution. In each of the FFPE and 18-tumour set samples, an *E. coli* lysate (reduced and alkylated) was added at this stage to monitor sample variability. To induce protein binding to the beads, lysate mixtures were adjusted to a final concentration of at least 50% acetonitrile (v/v). Bead-protein solutions were mixed to ensure a homogeneous distribution of the beads and incubated for a total of 8 minutes at room temperature. After incubation, tubes were placed on a magnetic rack for 2 minutes. While on the magnet, the supernatant was removed and discarded. The beads were rinsed twice through addition of 200 μL of freshly prepared 70% absolute ethanol, and the supernatant was discarded each time. Beads were then rinsed one further time with 180 μL of 100% acetonitrile, and the supernatant discarded. All rinses were carried out on the magnetic rack. Rinsed beads were reconstituted in aqueous buffer (~30 μL) and briefly sonicated in a water bath (30 seconds) to disaggregate the beads.

Detailed step-by-step protocols for SP3 sample handling can be found in the Supplemental Protocols.

### SP3-CTP Lysate (FFPE and frozen) Digest Preparation

For elution in the final stage of the SP3 protocol, the aqueous elution buffer consisted of 200 mM HEPES pH 8 with Trypsin/Lys-C mix (Promega) at an estimated 1:25 protein to enzyme ratio (μg/μg). A total of 30 μL of this enzyme-containing elution buffer was used per sample in all experiments. Bead-elution mixtures were sonicated for 30 seconds in a bath sonicator to disaggregate the SP3 beads. Samples were then incubated for 18-hours at 37 °C in a PCR thermocycler using the heated-lid option. After digestion, peptide-bead mixtures were sonicated for 30 seconds in a water bath. The eluted peptides were recovered using a magnetic rack and transferred to fresh tubes containing 20 μL of 200 mM HEPES pH 8 and the samples stored at −20 °C until TMT labeling. Representative samples were measured for peptide yield using UV spectrophotometry on a NanoDrop instrument (Thermo Scientific).

For tissue samples where a pooled internal standard was used, a 5 μL aliquot of every pre-TMT label digested sample (50 μL volume at this stage) was combined. From this pooled sample, 45 μL was used in the TMT labeling reaction as the 10^th^ channel. Detailed step-by-step protocols for tissue sample preparation can be found in the Supplemental Protocols.

### Mass Spectrometry Data Acquisition

Analysis of TMT labeled peptide fractions was carried out on an Orbitrap Fusion Tribrid MS platform (Thermo Scientific). Samples were introduced using an Easy-nLC 1000 system (Thermo Scientific). Columns used for trapping and analytical separations were packed in-house. Trapping columns were packed in 75 μm internal diameter capillaries to a length of 25 mm with C18 beads (Reprosil-Pur, Dr. Maisch, 3 μm particle size). Trap columns were fritted in-house using a combination of formamide and Kasil (1:3 ratio). Trapping was carried out for a total volume of 15 μL at a pressure of 400 bar. After trapping, gradient elution of peptides was performed on a C18 (Reprosil-Pur, Dr. Maisch, 3 μm particle size) column packed in-house in Pico-Frit (New Objective, 75 μm internal diameter) capillaries to a length of 50 cm and heated to 55 °C using AgileSLEEVE ovens (Analytical Sales & Service). Elution was performed with a gradient of mobile phase A (water and 0.1% formic acid) to 25% B (acetonitrile and 0.1% formic acid) over 100 minutes, and to 40% B over 20 minutes, with final elution (80% B) and equilibration (5% B) using a further 21 minutes at a flow rate of 350 nL/min.

Data acquisition on the Orbitrap Fusion (control software version 2.0.1258.15) was carried out using a data-dependent method with multi-notch synchronous precursor selection MS3 scanning for TMT tags. Survey scans covering the mass range of 350–1500 were acquired at a resolution of 120,000 (at m/z 200), with quadrupole isolation enabled, an S-Lens RF Level of 60%, a maximum fill time of 50 milliseconds, and an automatic gain control (AGC) target value of 4e5. For MS2 scan triggering, monoisotopic precursor selection was enabled, charge state filtering was limited to 2–5, an intensity threshold of 5e3 was employed, and dynamic exclusion of previously selected masses was enabled for 60 seconds with a tolerance of 10 ppm. MS2 scans were acquired in the ion trap in Turbo mode (scan rate 125,000, peak width at half height <3 Daltons) after CID fragmentation with a maximum fill time of 50 milliseconds, quadrupole isolation, an isolation window of 2 m/z, collision energy of 35%, activation Q of 0.25, injection for all available parallelizable time turned ON, and an AGC target value of 1e4. Fragment ions were selected for MS3 scans based on a precursor selection range of 400–1200 m/z, ion exclusion of 20 m/z low and 5 m/z high, and isobaric tag loss exclusion for TMT. The top 10 precursors were selected for MS3 scans that were acquired in the Orbitrap after HCD fragmentation (NCE 65%) with a maximum fill time of 120 milliseconds, 60,000 resolution, 110–750 m/z scan range, ion injection for all parallelizable time turned ON, and an AGC target value of 1e5. The total allowable cycle time was set to 4 seconds. MS1 and MS3 scans were acquired in profile mode, and MS2 in centroid format.

### Mass Spectrometry Data Analysis

Data from the Orbitrap Fusion were processed using Proteome Discoverer Software (ver. 2.1.0.62). MS2 spectra were searched using Sequest HT against a combined UniProt Human and *Escherichia coli* proteome database appended to a list of common contaminants (24,624 total sequences). Sequest HT parameters were specified as: trypsin enzyme, 2 missed cleavages allowed, minimum peptide length of 6, precursor mass tolerance of 20 ppm, and a fragment mass tolerance of 0.8 Daltons. Oxidation of methionine, and TMT at lysine and peptide N-termini were set as variable modifications. Carbamidomethylation of cysteine was set as a fixed modification. Peptide spectral match error rates were determined using the target-decoy strategy coupled to Percolator modeling of positive and false matches^56^,^57^. Reporter ions were quantified from MS3 scans using an integration tolerance of 20ppm with the most confident centroid setting. Output quantification values represented the signal-to-noise of the TMT value relative to the Orbitrap preamplifier. Data were filtered at the peptide spectral match-level to control for false discoveries using a q-value cut off of 0.05 as determined by Percolator. This less-stringent filter was applied to maximize sensitivity, relying on the statistical analyses during peptide quantification to further control for the potential generation of false conclusions within the final data set. As a result, the final quantitative set of hits that displays significant variance between tumour types is enriched in multi-peptide identified, high confidence proteins.

### Bioinformatic and Statistical Analyses

#### Proteomic data analysis

Data sets generated in Proteome Discoverer were exported and analyzed with a combination of scripts built in R designed in-house. Contaminant and decoy proteins were removed from all data sets prior to analysis. Unless stated otherwise, quantification was performed at the peptide level as discussed previously^58^,^59^. Briefly, peak areas and annotation information for unique peptides were combined into an expression set object and treated with a generalized-logarithm transformation using the VSN package^60^. In samples where the *E. coli* spike was used, peptide areas derived from the *E. coli* peptides were used to build an initial model of technical batch-to-batch variance using VSN that was then fit to the remaining human peptide values. Only *E. coli* peptides identified in all samples were used during this analysis. This VSN transformation addresses heterogeneity of variance across the dynamic range of peptide abundance. Statistical analysis of differential protein expression was performed at the peptide level using a modified version of the PECA function that is appropriate for input of log-transformed data^59^. PECA uses Limma^61^ to generate a linear model for estimating fold changes and standard errors prior to empirical Bayes smoothing. Median t-statistics of the assigned peptides are used to calculate false-discovery rate adjusted *p*-values, that are determined from the beta distribution as described previously^59^.

#### Human Protein Atlas data analysis

Data for protein expression values derived from the Human Protein Atlas (HPA)^25^ were downloaded from the main resource website. The categorical measurements provided in the HPA for each protein are a collection from multiple individual tissue blocks. Expression values were calculated based on assigning a numerical score of 9 for ‘High’, 6 for ‘Medium’, 3 for ‘Low’, and 0 for ‘Not detected’. To generate a single value per protein across all tissue blocks, each value was multiplied by the number of blocks assigned to that class (e.g. ‘High’, ‘Medium’, ‘Low’, ‘Not detected’) and the total sum per protein calculated. The resulting value is a rough estimate of expression based on the numbers of tissue blocks assigned in each category by the HPA group. Ovarian carcinoma analyses were not differentiated based on histotype in the HPA, and thus contains values for HGSC, CCC, ENOC, and Mucinous classes.

#### RNA expression analysis

RNA expression data were downloaded from the gene expression omnibus (GEO) for the accession: GSE65986^30^. This data consists of microarray-derived measurements of gene expression from 55 ovarian carcinomas (25 CCCs, 16 HGSC, and 14 ENOCs). The extracted CEL files underwent background subtraction, normalization, and log transformation using gcrma in R^62^. Differential expression was determined using the moderated t-test with empirical Bayes smoothing within Limma as with the protein data. Probes were further condensed into single measurements based on the median of all values assigned per gene. TCGA-based FPKM RNA expression values were accessed using the R entry point from cBioPortal^63^.

#### Methylation and Prognosis marker analysis

Values for promoter methylation were taken from the TCGA ovarian carcinoma data^19^. Genes were queried using the R entry point from cBioPortal. Prognosis feature analysis was done using a published set of genes derived from RNA expression analysis^19^.

#### Gene set enrichment analysis

Global comparisons of enriched gene sets and ontologies independent of expression was performed using Metascape^31^. Gene lists consisting of proteins having a log2 fold-change above 0.5 and an adjusted p-value below 0.05 were compiled for each histotype relative to the two others. Gene lists were queried against biological process, molecular function, and cellular component gene ontologies, and the Hallmark signature set from MSigDB^32^.

To capture enrichment of gene sets where protein expression was considered (GSEA), analysis was performed manually using sets derived from the MSigDB resource^32^. Gene collections were imported into R and profiled using an in-house script that calculates the mean difference in expression of a gene set in relation to total expression. Statistical enrichment analysis was performed using the geneSetTest function built into Limma^64^. Statistics utilized in the geneSetTest function were calculated based on relative log2 expression values for each histotype (relative to the median per gene). The derivation of t-statistics on a per gene basis was performed using the moderated t-test in Limma^61^. Only sets where a minimum of 50% of the total set had representative values in our own data were considered in this analysis.

#### Gene ontology analysis

Gene ontology assignment and enrichment analysis was performed using g:Profiler^65^. The g:GOSt tool in the g:Profiler suite uses a Fisher’s one-tailed test to measure the statistical significance of enrichment for any given GO term. Multiple testing is corrected using the built-in g:SCS method, as described previously^65^.

#### Immunohistochemistry and scoring

Tissue microarrays (TMAs) were constructed as duplicate cores of FFPE materials from 485 primary ovarian epithelial, sex cord stromal and germ cell tumours. 4 μm sections of the TMAs on Superfrost + glass slides were processed using the Ventana Discovery XT, and the Ventana Benchmark XT and Benchmark Ultra automated systems (Ventana Medical Systems). TMAs were stained with an antibody to CTH (1:250, clone 1E12; LS-C337259 LSBio). The TMA images were scored by two pathologists (TN and ANK). Tumours were scored as negative if there was an absence of staining in the epithelial cells. A score of 1 was given if there was diffuse (>70% of the cells), weak staining or the staining was variable. A score of 2 indicates diffuse strong staining. If the duplicate cores were given different scores, an overall score of 1, indicating variable staining, was given.

#### Western blotting

Whole protein extract was prepared in RIPA lysis buffer and 30 mg of protein was separated by SDS-PAGE electrophoresis and transferred to nitrocellulose membranes. Blots were probed with antibodies to CTH (1:1000, clone E12, LS-C337259 LSBio), LEFTY1 (1:1000, LS-B5830 LSBio) and β-Actin (1:10,000, clone 8H10D10, Cell Signaling Technology), followed by probing with peroxidase conjugated secondary antibodies raised in goat against rabbit or mouse (1:10,000, ABM Inc). The signal was detected by chemiluminescence (Millipore).

### Data and Code Availability

The mass spectrometry proteomics data have been deposited to the ProteomeXchange Consortium^66^ via the PRIDE^67^ partner repository with the dataset identifier PXD003607. Identification results, MS acquisition methods, Proteome Discoverer workflows, sequence databases, are also stored on ProteomeXChange under the same identifier. Processed peptide data sets are provided in ProteomeXChange as R data storage objects to avoid transformation of gene identifiers in external software. The contents of each storage object are described in Supplemental Table 6. R code used to perform all analyses is openly available on GitHub (https://github.com/chrishuges/OvC) and in the ProteomeXChange repository. The contents of each R session are described in Supplemental Table 7. Supplementary code for extracting the data from an R data storage object is available in the Supplemental Protocols. Descriptions of naming conventions used in the data file, or analysis file names are provided in Supplemental Table 8. Any additional files are openly available upon request.

## Additional Information

**How to cite this article**: Hughes, C. S. *et al*. Quantitative Profiling of Single Formalin Fixed Tumour Sections: proteomics for translational research. *Sci. Rep.* **6**, 34949; doi: 10.1038/srep34949 (2016).

## Supplementary Material

Supplementary Information

Supplementary Information

## References

[b1] Sallam R. M. Proteomics in Cancer Biomarkers Discovery: Challenges and Applications. Dis. Markers 2015, 12 (2015). 10.1155/2015/321370 PMC4427011 25999657

[b2] Gustafsson O. J. R., Arentz G. & Hoffmann P. Proteomic developments in the analysis of formalin-fixed tissue. Biochim. Biophys. Acta - Proteins Proteomics 1854, 559–580 (2015). 10.1016/j.bbapap.2014.10.003 25315853

[b3] Zhang B. . Proteogenomic characterization of human colon and rectal cancer. Nature 513, 382–387 (2014). 25043054 10.1038/nature13438 PMC4249766

[b4] Mertins P. . Proteogenomics connects somatic mutations to signalling in breast cancer. Nature 1–19, doi: 10.1038/nature18003 (2016). PMC5102256 27251275

[b5] Zhang H. . Integrated Proteogenomic Characterization of Human High-Grade Serous Ovarian Cancer. Cell, doi: 10.1016/j.cell.2016.05.069 (2016). PMC4967013 27372738

[b6] Tyanova S. . Proteomic maps of breast cancer subtypes. Nat. Commun. 7, 10259 (2016). 26725330 10.1038/ncomms10259 PMC4725767

[b7] Pozniak Y. . System-wide Clinical Proteomics of Breast Cancer Reveals Global Remodeling of Tissue Homeostasis. Cell Syst. 2, 172–184 (2016). 27135363 10.1016/j.cels.2016.02.001

[b8] Wisniewski J. R., Ostasiewicz P. & Mann M. High recovery FASP applied to the proteomic analysis of microdissected formalin fixed paraffin embedded cancer tissues retrieves known colon cancer markers. J. Proteome Res. 10, 3040–3049 (2011). 21526778 10.1021/pr200019m

[b9] Wiśniewski J. R. . Extensive quantitative remodeling of the proteome between normal colon tissue and adenocarcinoma. Mol. Syst. Biol. 8, 611 (2012). 22968445 10.1038/msb.2012.44 PMC3472694

[b10] Wiśniewski J. R. . Absolute Proteome Analysis of Colorectal Mucosa, Adenoma, and Cancer Reveals Drastic Changes in Fatty Acid Metabolism and Plasma Membrane Transporters. J. Proteome Res. 14, 4005–4018 (2015). 26245529 10.1021/acs.jproteome.5b00523

[b11] Quesada-Calvo F. . Comparison of two FFPE preparation methods using label-free shotgun proteomics: Application to tissues of diverticulitis patients. J. Proteomics 112C, 250–261 (2014). 10.1016/j.jprot.2014.08.013 25218866

[b12] Wisniewski J. R., Zielinska D. F. & Mann M. Comparison of ultrafiltration units for proteomic and N-glycoproteomic analysis by the filter-aided sample preparation method. Anal. Biochem. 410, 307–309 (2011). 21144814 10.1016/j.ab.2010.12.004

[b13] Shih I.-M. & Kurman R. J. Ovarian Tumorigenesis. Am. J. Pathol. 164, 1511–1518 (2004). 15111296 10.1016/s0002-9440(10)63708-x PMC1615664

[b14] Bell D. a. Origins and molecular pathology of ovarian cancer. Mod. Pathol. 18 Suppl 2, S19–S32 (2005). 15761464 10.1038/modpathol.3800306

[b15] Köbel M. . Ovarian carcinoma subtypes are different diseases: Implications for biomarker studies. PLoS Med. 5, 1749–1760 (2008). 10.1371/journal.pmed.0050232 PMC2592352 19053170

[b16] Köbel M. . Diagnosis of ovarian carcinoma cell type is highly reproducible: a transcanadian study. Am. J. Surg. Pathol. 34, 984–993 (2010). 20505499 10.1097/PAS.0b013e3181e1a3bb

[b17] Kurman R. J. & Shih I. M. Molecular pathogenesis and extraovarian origin of epithelial ovarian cancer - Shifting the paradigm. Human Pathology 42, 918–931 (2011). 21683865 10.1016/j.humpath.2011.03.003 PMC3148026

[b18] Konstantinopoulos P. a, Spentzos D. & Cannistra S. a. Gene-expression profiling in epithelial ovarian cancer. Nat. Clin. Pract. Oncol. 5, 577–587 (2008). 18648354 10.1038/ncponc1178

[b19] TCGA. Integrated genomic analyses of ovarian carcinoma. Nature 474, 609–615 (2011). 21720365 10.1038/nature10166 PMC3163504

[b20] Elzek M. a. & Rodland K. D. Proteomics of ovarian cancer: functional insights and clinical applications. Cancer Metastasis Rev. 34, 83–96 (2015). 25736266 10.1007/s10555-014-9547-8 PMC4776756

[b21] Albert F. W. & Kruglyak L. The role of regulatory variation in complex traits and disease. Nat. Rev. Genet. 16, 197–212 (2015). 25707927 10.1038/nrg3891

[b22] Hughes C. S. . Ultrasensitive proteome analysis using paramagnetic bead technology. Mol. Syst. Biol. 10, 1–14 (2014). 10.15252/msb.20145625 PMC4299378 25358341

[b23] Thompson A. . Tandem mass tags: a novel quantification strategy for comparative analysis of complex protein mixtures by MS/MS. Anal. Chem. 75, 1895–1904 (2003). 12713048 10.1021/ac0262560

[b24] Shi S.-R., Liu C., Balgley B. M., Lee C. & Taylor C. R. Protein extraction from formalin-fixed, paraffin-embedded tissue sections: quality evaluation by mass spectrometry. J. Histochem. Cytochem. 54, 739–743 (2006). 16399996 10.1369/jhc.5B6851.2006

[b25] Uhlen M. . Tissue-based map of the human proteome. Science (80-.). 347, 1260419–1260419 (2015). 10.1126/science.1260419 25613900

[b26] Köbel M. . A limited panel of immunomarkers can reliably distinguish between clear cell and high-grade serous carcinoma of the ovary. Am. J. Surg. Pathol. 33, 14–21 (2009). 18830127 10.1097/PAS.0b013e3181788546

[b27] Zorn K. K. Gene Expression Profiles of Serous, Endometrioid, and Clear Cell Subtypes of Ovarian and Endometrial Cancer. Clin. Cancer Res. 11, 6422–6430 (2005). 16166416 10.1158/1078-0432.CCR-05-0508

[b28] Spentzos D. Gene Expression Signature With Independent Prognostic Significance in Epithelial Ovarian Cancer. J. Clin. Oncol. 22, 4700–4710 (2004). 15505275 10.1200/JCO.2004.04.070

[b29] Schwartz D. R. . Gene Expression in Ovarian Cancer Reflects Both Morphology and Biological Behavior. Distinguishing Clear Cell from Other Poor-Prognosis Ovarian Carcinomas Gene Expression in Ovarian Cancer Reflects Both Morphology and Biological. 4722–4729 (2002). 12183431

[b30] Uehara Y. . Integrated Copy Number and Expression Analysis Identifies Profiles of Whole-Arm Chromosomal Alterations and Subgroups with Favorable Outcome in Ovarian Clear Cell Carcinomas. PLoS One 10 (2015). 10.1371/journal.pone.0128066 PMC4456367 26043110

[b31] Tripathi S. . Meta- and Orthogonal Integration of Influenza ‘OMICs’ Data Defines a Role for UBR4 in Virus Budding. Cell Host Microbe 18, 723–735 (2015). 26651948 10.1016/j.chom.2015.11.002 PMC4829074

[b32] Subramanian A. . Gene set enrichment analysis: a knowledge-based approach for interpreting genome-wide expression profiles. Proc. Natl. Acad. Sci. USA 102, 15545–15550 (2005). 16199517 10.1073/pnas.0506580102 PMC1239896

[b33] Yamaguchi K. . Identification of an ovarian clear cell carcinoma gene signature that reflects inherent disease biology and the carcinogenic processes. Oncogene 29, 1741–1752 (2010). 20062075 10.1038/onc.2009.470

[b34] Pontén F. . A global view of protein expression in human cells, tissues, and organs. Mol. Syst. Biol. 5, 337 (2009). 20029370 10.1038/msb.2009.93 PMC2824494

[b35] de Sousa Abreu R., Penalva L. O., Marcotte E. M. & Vogel C. Global signatures of protein and mRNA expression levels. Mol. Biosyst. 5, 1512–1526 (2009). 20023718 10.1039/b908315d PMC4089977

[b36] Maier T., Güell M. & Serrano L. Correlation of mRNA and protein in complex biological samples. FEBS Lett. 583, 3966–3973 (2009). 19850042 10.1016/j.febslet.2009.10.036

[b37] Vogel C. & Marcotte E. M. Insights into the regulation of protein abundance from proteomic and transcriptomic analyses. Nat. Rev. Genet. 13, 227–232 (2012). 22411467 10.1038/nrg3185 PMC3654667

[b38] Vergote I. B., Marth C. & Coleman R. L. Role of the folate receptor in ovarian cancer treatment: evidence, mechanism, and clinical implications. Cancer Metastasis Rev. 34, 41–52 (2015). 25564455 10.1007/s10555-014-9539-8

[b39] Köbel M. . Evidence for a time-dependent association between FOLR1 expression and survival from ovarian carcinoma: implications for clinical testing. An Ovarian Tumour Tissue Analysis consortium study. Br. J. Cancer 111, 2297–2307 (2014). 25349970 10.1038/bjc.2014.567 PMC4264456

[b40] Dhanoa B. S., Cogliati T., Satish A. G., Bruford E. A. & Friedman J. S. Update on the Kelch-like (KLHL) gene family. Hum. Genomics 7, 13 (2013). 23676014 10.1186/1479-7364-7-13 PMC3658946

[b41] Márquez A. . Effect of BSN-MST1 locus on inflammatory bowel disease and multiple sclerosis susceptibility. Genes Immun. 10, 631–635 (2009). 19657358 10.1038/gene.2009.56

[b42] Sasaki A. . Difference in mesothelin-binding ability of serum CA125 between patients with endometriosis and epithelial ovarian cancer. Int. J. Cancer 136, 1985–1990 (2015). 25197000 10.1002/ijc.29185

[b43] O’Shannessy D. J. . Serum folate receptor alpha, mesothelin and megakaryocyte potentiating factor in ovarian cancer: association to disease stage and grade and comparison to CA125 and HE4. J. Ovarian Res. 6, 29 (2013). 23590973 10.1186/1757-2215-6-29 PMC3640997

[b44] Tabibzadeh S. & Hemmati-Brivanlou A. Lefty at the crossroads of ‘stemness’ and differentiative events. Stem Cells 24, 1998–2006 (2006). 16728558 10.1634/stemcells.2006-0075

[b45] Postovit L.-M., Seftor E. a, Seftor R. E. B. & Hendrix M. J. C. Targeting Nodal in malignant melanoma cells. Expert Opin. Ther. Targets 11, 497–505 (2007). 17373879 10.1517/14728222.11.4.497

[b46] Quail D. F. . Embryonic protein nodal promotes breast cancer vascularization. Cancer Res. 72, 3851–3863 (2012). 22855743 10.1158/0008-5472.CAN-11-3951

[b47] Quail D. F. . Embryonic Morphogen Nodal Promotes Breast Cancer Growth and Progression. PLoS One 7 (2012). 10.1371/journal.pone.0048237 PMC3492336 23144858

[b48] Quail D. F., Zhang G., Findlay S. D., Hess D. a. & Postovit L.-M. Nodal promotes invasive phenotypes via a mitogen-activated protein kinase-dependent pathway. Oncogene 33, 461–473 (2014). 23334323 10.1038/onc.2012.608 PMC5025281

[b49] Hughes C. . Mass spectrometry-based proteomic analysis of the matrix microenvironment in pluripotent stem cell culture. Mol. Cell. Proteomics 11, 1924–1936 (2012). 23023296 10.1074/mcp.M112.020057 PMC3518136

[b50] Postovit L.-M. . Human embryonic stem cell microenvironment suppresses the tumorigenic phenotype of aggressive cancer cells. Proc. Natl. Acad. Sci. USA 105, 4329–4334 (2008). 18334633 10.1073/pnas.0800467105 PMC2393795

[b51] Wang R. Two’s company, three’s a crowd: can H2S be the third endogenous gaseous transmitter? FASEB J. 16, 1792–1798 (2002). 12409322 10.1096/fj.02-0211hyp

[b52] Bhattacharyya S. . Cystathionine Beta-Synthase (CBS) Contributes to Advanced Ovarian Cancer Progression and Drug Resistance. PLoS One 8 (2013). 10.1371/journal.pone.0079167 PMC3827285 24236104

[b53] Mandai M. . Ovarian clear cell carcinoma meets metabolism; HNF-1β confers survival benefits through the Warburg effect and ROS reduction. Oncotarget 6, 30704–14 (2015). 26375553 10.18632/oncotarget.5228 PMC4741562

[b54] Tsuchiya A. . Expression Profiling in Ovarian Clear Cell Carcinoma. Am. J. Pathol. 163, 2503–2512 (2003). 14633622 10.1016/s0002-9440(10)63605-x PMC1892387

[b55] Yamamoto S. . Immunohistochemical detection of hepatocyte nuclear factor 1β in ovarian and endometrial clear-cell adenocarcinomas and nonneoplastic endometrium. Hum. Pathol. 38, 1074–1080 (2007). 17442376 10.1016/j.humpath.2006.12.018

[b56] Käll L., Canterbury J. D., Weston J., Noble W. S. & MacCoss M. J. Semi-supervised learning for peptide identification from shotgun proteomics datasets. Nat. Methods 4, 923–925 (2007). 17952086 10.1038/nmeth1113

[b57] Spivak M. . Improvements to the Percolator Algorithm for Peptide Identi cation from Shotgun Proteomics Data Sets. J. Proteome Res. 8, 3737–3745 (2009). 19385687 10.1021/pr801109k PMC2710313

[b58] Karp N. a. . Addressing accuracy and precision issues in iTRAQ quantitation. Mol. Cell. Proteomics 9, 1885–1897 (2010). 20382981 10.1074/mcp.M900628-MCP200 PMC2938101

[b59] Suomi T., Corthals G. L., Nevalainen O. S. & Elo L. L. Using Peptide-Level Proteomics Data for Detecting Differentially Expressed Proteins. J. Proteome Res. 14, 4564–4570 (2015). 26380941 10.1021/acs.jproteome.5b00363

[b60] Huber W., von Heydebreck A., Sültmann H., Poustka A. & Vingron M. Variance stabilization applied to microarray data calibration and to the quantification of differential expression. Bioinformatics 18 Suppl 1, S96–S104 (2002). 12169536 10.1093/bioinformatics/18.suppl_1.s96

[b61] Smyth G. K. Linear models and empirical bayes methods for assessing differential expression in microarray experiments. Stat. Appl. Genet. Mol. Biol. 3 (2004). 10.2202/1544-6115.1027 16646809

[b62] Wu Z., Irizarry R. a., Gentleman R., Martinez-Murillo F. & Spencer F. A Model-Based Background Adjustment for Oligonucleotide Expression Arrays. J. Am. Stat. Assoc. 99, 909–917 (2004).

[b63] Cerami E. . The cBio Cancer Genomics Portal: An open platform for exploring multidimensional cancer genomics data. Cancer Discov. 2, 401–404 (2012). 22588877 10.1158/2159-8290.CD-12-0095 PMC3956037

[b64] Goeman J. J. & Bühlmann P. Analyzing gene expression data in terms of gene sets: Methodological issues. Bioinformatics 23, 980–987 (2007). 17303618 10.1093/bioinformatics/btm051

[b65] Reimand J., Kull M., Peterson H., Hansen J. & Vilo J. g:Profiler–a web-based toolset for functional profiling of gene lists from large-scale experiments. Nucleic Acids Res. 35, W193–W200 (2007). 17478515 10.1093/nar/gkm226 PMC1933153

[b66] Vizcaíno J., Deutsch E. & Wang R. ProteomeXchange provides globally coordinated proteomics data submission and dissemination. Nat. … 32, 223–226 (2014). 10.1038/nbt.2839 PMC3986813 24727771

[b67] Vizcaíno J. A. . 2016 update of the PRIDE database and its related tools. Nucleic Acids Res. 44, D447–D456 (2016). 26527722 10.1093/nar/gkv1145 PMC4702828

