# Supplementary material for: Quantitative Profiling of Single Formalin Fixed Tumour Sections: proteomics for translational research
Source: Sci Rep. 2016 Oct 7;6:34949. doi: 10.1038/srep34949 (PMC5054533; doi:10.1038/srep34949)

Supplemental Information for: Quantitative Profiling of Single Formalin Fixed Tumour Sections: proteomics for translational research

Christopher S Hughes[1,¶], Melissa K McConechy[2,¶], Dawn R Cochrane[4], Tayyebeh Nazeran[3], Anthony N Karnezis[3], David G Huntsman[3,4], Gregg B Morin[1,5,*]

1 – Canada's Michael Smith Genome Sciences Centre, British Columbia Cancer Agency, Vancouver, British Columbia, Canada

2 – Department of Human Genetics, Research Institute of the McGill University Health Network, McGill University, Montreal, Canada

3– Department of Pathology and Laboratory Medicine, University of British Columbia, British Columbia Cancer Agency, Vancouver, British Columbia, Canada

4 – Department of Molecular Oncology, British Columbia Cancer Agency, Vancouver, British Columbia, Canada

5 – Department of Medical Genetics, University of British Columbia, Vancouver, British Columbia, Canada

¶ – these authors contributed equally to this work

* To whom correspondence should be addressed: gmorin@bcgsc.ca

Supplemental Table of Contents

Supplemental Methods 3
Paramagnetic Beads 3
Cell culture and harvest 3
Cell line lysis, protein reduction and alkylation 3
E. coli standard preparation 4
Cell line lysate digest preparation 5
Tandem mass tag labelling of peptides 5
Peptide clean-up procedures 6
High-pH reversed phase fractionation 6
Supplemental Protocols 8
Reagents & Equipment 8
Protocol: Tissue lysis (version used in this manuscript) 8
Protocol: SP3 protein clean up (version used in this manuscript) 9
Protocol: TMT peptide labelling (version used in this manuscript) 11
Protocol: Extracting data files from a .RDS object 12
Protocol: SP3-CTP Complete (current optimized version of the protocol used by the author group) 13
Supplemental References 20
Supplemental Figure Legends 21
Supplemental Tables 30

Supplemental Methods

Paramagnetic Beads

In all experiments with SP3 the beads used were a 1:1 mix of beads with a hydrophilic (Sera-Mag Speed Beads, GE Life Sciences, CAT# 45152105050350) or hydrophobic (CAT# 65152105050350) surface. The magnetic particles are an average diameter of 1µm. Beads come from the manufacturer at a concentration of ~50 mg/mL. Magnetic racks used in all experiments were designed and manufactured in-house as described previously [1].

Cell culture and harvest

OVCAR-3, OVCAR-5, OVSAHO, JHOC-5, JHOC7, OVISE, OVMANA, Kuramochi, Hey, ES2, A2780, IGROV1 and OVTOKO cells were cultured in RPMI supplemented with 10% fetal bovine serum (FBS). CaOV3, TOV21G, VOA295 and TOV112D were cultured in 199/105 supplemented with 10% FBS. Cells were maintained at 37°C in a 5% CO_2 environment. Cells were harvested through incubation with a solution of 0.05% trypsin-EDTA, and centrifuged. Recovered cells were rinsed and counted prior to aliquoting to the desired cell number per tube. Approximate cell counts were acquired using a haemocytometer. Cell line integrity was assessed using short-tandem repeat analysis described previously [2].

Cell line lysis, protein reduction and alkylation

Frozen pellets derived from cell lines were lysed using a solution-based procedure without physical disruption. Lysis buffer was composed of 1% SDS

(Bio-Rad), 1X cOmplete Protease Inhibitor Cocktail-EDTA (Roche), prepared in 50mM HEPES buffer at pH 8.5 (Sigma). Lysis was induced through addition of a volume of lysis buffer equal to the volume occupied by the pellet (~75 µL). Mixtures were heated for 5 minutes at 95°C and subsequently placed on ice for 5 minutes. To each tube, 25 Units of Benzonase Nuclease (EMD Millipore, >99% purity) was added per estimated 500,000 cells to degrade chromatin. Tubes were incubated at 37°C for 30 minutes in a thermocycler. Proteins in the lysates were reduced and alkylated through the addition of 10 mM TCEP (Sigma) and 40 mM chloroacetamide (Sigma)(final concentrations) with incubation at 37°C for 30 minutes. Lysates were stored at -80°C until use.

***E. coli* standard preparation**

DH5-alpha *E. coli* (Thermo Fisher, CAT#18258012) were grown in LB broth in 100 mL flask cultures in standard conditions overnight to achieve stationary phase growth. Each 100 mL culture was centrifuged and excess medium removed and pellets rinsed 1X in PBS. Lysis of a pellet derived from 100 mL of *E. coli* culture was carried out using 2 mL of 4% SDS with 5 mM dithiothreitol (Sigma) in 50mM HEPES pH 7.5. Mixtures were heated at 95°C for 10 minutes, and sonicated using a Bioruptor Pico device (Diagenode, 15 cycles, 30 seconds on, 30 seconds off). The sonicated lysate was incubated for a further 30 minutes at 37°C to ensure disulfide reduction with dithiothreitol (DTT), and iodoacetamide (IAA) added for alkylation with incubation at room temperature for 60 minutes. After quenching with dithiothreitol, 10 µL, ~250 Units of Benzonase was added and the sample incubated at 37°C for 30 minutes. The

prepared lysate was then precipitated through addition of 4 volumes of ice-cold acetone and overnight incubation at -20°C. The next day, samples were spun at 500g to pellet the insoluble material, and the supernatant discarded. Pellets were rinsed twice with ice-cold acetone, and later reconstituted in 5mL of a solution of 1% SDS in 200mM HEPES pH 7.5. Protein concentration was measured using a BCA assay (Thermo Scientific) and aliquots were stored at -80°C until use.

Cell line lysate digest preparation

Prepared cell-line lysates that had been reduced and alkylated were treated with SP3 as described previously [1] (Supplemental Protocols). SP3-treated lysates were eluted in 30 µL of an buffer consisting of 200mM HEPES pH 8 with Trypsin/Lys-C mix (Promega) at an estimated 1:25 protein to enzyme ratio (µg/µg). Bead-elution mixtures were sonicated for 30 seconds in a bath sonicator to disaggregate the SP3 beads. Samples were incubated for 18-hours at 37°C in a PCR thermocycler using the heated-lid option. After digestion, peptide-bead mixtures were sonicated for 30 seconds in a water bath to help disaggregate the beads. Peptides were recovered from the magnetic beads and transferred to fresh tubes containing 20 µL of 200mM HEPES pH 8 and the samples stored at -20°C until TMT labeling.

Tandem mass tag labelling of peptides

TMT 10- and 6-plex labeling kits were obtained from Pierce. Each TMT label (5 mg per vial) was reconstituted in 500 µL of acetonitrile and refrozen. Peptide solutions derived from SP3 digests were typically contained in a total volume of

50 µL of digestion buffer (0.2M HEPES pH 8). Labeling reactions were carried out through addition of 100 µg of TMT label in two volumetrically equal steps of 5 µL (50 µg per addition), 30 minutes apart. TMT labeling reactions were carried out after the removal of peptide digests to fresh tubes from the magnetic beads. Reactions were quenched through addition of 10 µL of 1M glycine (Sigma). Labeled peptides were combined and treated with SepPak clean-up prior to HPLC fractionation.

Peptide clean-up procedures

Peptides were desalted and concentrated using either SepPak or StageTip treatment. For SepPak clean-up, cartridges (50 mg C18-t, Waters) were rinsed twice with 1mL of acetonitrile with 0.1% formic acid. Cartridges were then rinsed twice with 1mL of water with 0.1% formic acid prior to sample loading. Loaded samples were rinsed twice with 0.1% formic acid and eluted with 1 mL of 80% acetonitrile containing 0.1% formic acid. StageTips were prepared as previously described [3]. StageTips were rinsed and eluted using the same conditions as with SepPak cartridges. In both cases, eluted samples were concentrated in a SpeedVac centrifuge (Thermo Scientific) and subsequently reconstituted in 0.1% formic acid.

High-pH reversed phase fractionation

High-pH reversed phase analysis was performed on an Agilent 1100 HPLC system equipped with a diode array detector (254, 260, and 280nm). Fractionation was performed on a Kinetix EVO C18 column (2.1 x 150mm, 1.7 µm core shell, 100Å, Phenomenex). Elution was performed at a flow rate of

0.1mL per minute using a gradient of mobile phase A (20mM ammonium hydroxide, pH 10) and B (acetonitrile), from 1% to 35% over 90 minutes. Fractions were collected every minute across the entire sample run and gradient length (96 total fractions) and concatenated into 10 or 12 final samples as discussed previously [4]. Fractions were dried in a SpeedVac centrifuge and reconstituted in 1% formic acid prior to MS analysis.

Supplemental Protocols

To ensure the complete reproducibility of our work, any detailed step-by-step protocols beyond those described here are openly available upon request from the authors.

Reagents & Equipment

- Sera-Mag Speed Beads A: (GE Life Sciences; CAT# 45152105050350, Magnetic Carboxylate Modified).
- Sera-Mag Speed Beads B: (GE Life Sciences; CAT# 65152105050350, Magnetic Carboxylate Modified).
- Benzonase nuclease (CAT# 101697, EMD Millipore)
- Magnetic stand: needs to be capable of holding PCR tubes. Racks used are manufactured as described previously [1].
- 10X TCEP and CAA solution (100mM TCEP - CAT# C4706, Sigma and 400mM Chloroacetamide - CAT# C0267, Sigma)
- 1M Glycine (CAT# G8898, Sigma)
- Trypsin/Lys-C Mix (CAT# V5071, Promega)
- Tandem Mass Tag Labeling Kit (CAT# 90111, Thermo Fisher)

Protocol: Tissue lysis (version used in this manuscript)

Critical: The steps described here are based on the use of a single 10µm section from an FFPE tissue block (~1cm X ~1.5cm) that has been de-parafinized with xylene and rinsed with ethanol.

1. Add 30 µL of nuclease digestion solution (1% SDS and 100U of Benzonase in 0.2M HEPES pH 8) to the tissue section, and incubate at 37°C for 1 hour. **Note**: the tissue will remain largely insoluble at this point, and mostly intact.
2. Add 30 µL of 20% SDS and mix.
3. Heat the tissue section for 45 minutes at 95°C to remove formalin crosslinks.
4. Add 6.6 µL of the 10X TCEP and chloroacetamide solution and incubate for 30 minutes at 37°C (10mM TCEP and 40mM chloroacetamide final).
5. Prepared lysates can be stored frozen or taken directly to SP3 protein clean up.

Protocol: SP3 protein clean up (version used in this manuscript)

Critical: The steps described here are based on the use of lysate from the tissue preparation protocol. The volume is assumed to be ~70 µL based on the previous protocols.

1. Add 5 µL of each of the SP3 bead types and mix.
2. Add acetonitrile to obtain a final percentage of at least 50% (80 µL of a 100% stock was used).
3. Incubate for 8 minutes at room temperature off the rack. **Note**: you should observe bead clumping and clusters forming as the incubation progresses. There may be a small amount of insoluble material that remains after the lysis and solubilization processing. This will be carried through into the

digestion protocol through interaction with the SP3 beads, and will be brought into solution through digestion.

4. Place on magnetic rack and incubate for further 2 minutes at room temperature.

5. Remove and discard supernatant.

6. Add 200 µL of 70% ethanol and incubate for 30 seconds on magnetic stand. Remove and discard supernatant. **Note**: it is a good idea here to pipette the rinse up and down a couple of times, and aspirate onto the beads that are bound to the tube wall by the magnet. The concentration of SDS is high, and performing more aggressive rinsing of the beads can help remove this detergent completely. Avoid removing the tubes from the magnet during rinsing, as this will result in significant sample loss.

6. Add 200 µL of 70% ethanol and incubate for 30 seconds on magnetic stand. Remove and discard supernatant.

7. Add 180 µL of acetonitrile and incubate for 15 seconds on magnetic stand. Remove and discard supernatant and air-dry the beads for 30 seconds.

8. Reconstitute beads in water (or desired buffer of choice). For digestion, reconstitute beads in 30 µL of digestion solution (e.g. 200 mM HEPES pH 8 + X µg of trypsin/lysC mix (1:25 enzyme to substrate ratio)). **Note**: the beads will be very sticky at this point, and it is best to minimize their handling. Typically, it is best to gently push the beads into the digestion liquid off the side of the tube using the tip of a pipette tip, without actually

pipetting them. If the beads are pipetted, they can easily stick to the inside of the pipette tip. Sonicate the beads for 30 seconds in a water bath (or until you can see they are disaggregated, usually just a couple of seconds), and then proceed with pipetting to ensure complete mixing.

9. Incubate for 14 hours at 37°C.

Digested peptides can be recovered from the beads by placing the tube on a magnetic rack and removing the supernatant. The supernatant will contain the eluted peptides. The peptide mixture can be used directly in downstream fractionation, labeling, or conventional clean-up workflows. The beads can be sonicated in a water bath to improve recovery of the peptides.

Protocol: TMT peptide labelling (version used in this manuscript)

Critical: To prepare the TMT label stock, reconstitute the original 5 mg label in 500 µL of acetonitrile (10 µg/µL final) and vortex mix. To store the unused label, it can be aliquoted and kept at -80°C. Alternatively, the original tube containing the 500 µL of stock can be wrapped in parafilm, and stored at -80°C and thawed multiple times for use. We have undertaken >20 cycles of freeze/thaw with a single TMT tube with no loss in label fidelity.

1. Transfer the 30 µL of digested peptides without the beads from the SP3 protein clean-up protocol to a fresh 1.5 mL eppendorf tube containing 20 µL of 200mM HEPES pH 8.

2. Add 5 µL of TMT label to the corresponding tubes and pipette mix. Incubate at room temperature for 30 minutes.

3. Add an additional 5 μL of TMT label to the tubes and pipette mix. Incubate at room temperature for 30 minutes.
4. Add 10 μL of 1M glycine to each tube and pipette mix. Incubate for 30 minutes at room temperature.
5. Combine the TMT labeled samples and SpeedVac for 1.5 hours to remove residual acetonitrile.
6. Add 0.1% formic acid to the sample to a final volume of 700 μL. The sample can now be cleaned up using a SepPak prior to fractionation.

Protocol: Extracting data files from a .RDS object

Critical: R needs to be pre-installed on the analysis computer to perform this task. Any version of R is fine - we used R version 3.2.2. (rel. 2015-08-14) – "Fire Safety".

Paste this code in your R console:

```
>setwd(dir="/directory/where/you/saved/the/rds/file")

>x = readRDS("nameoftheRDSfile.rds")

>write.table(x,'desiredfilename.txt',quote=FALSE,sep='\t',col.names=TRUE,
row.names=FALSE)
```

Protocol: SP3-CTP Complete (current optimized version of the protocol used by the author group)

Notes:

- This protocol assumes you are working in a 10-plex experiment using a pooled standard control with spiked *E. coli* proteins.
- This protocol works optimally 10um scrolls of tissue (~1cm X ~1cm).
- Take note of whether the sample has been deparaffinized or not.

Materials:

- 20% SDS (stock solution prepared from solid SDS) (Bio-Rad, CAT#1610302)
- 0.2M HEPES pH 8.5 (Sigma, CAT#H3375)
- 0.2M HEPES pH 8 (Sigma, CAT#H3375)
- Benzonase nuclease (EMD Millipore, CAT#70664)
- 200mM DTT (15mg of dithiothreitol in 500uL 0.2M HEPES pH 8.5) - prepare fresh! (Bio-Rad, CAT#1610611)
- 400mM IAA (36mg of iodoacetamide in 500uL 0.2M HEPES pH 8.5) - prepare fresh! (Bio-Rad, CAT#1632109)
- SP3 Beads (GE Healthcare, CAT#45152105050250, 65152105050250)
- Acetonitrile (Sigma, CAT#271004)
- Ethanol (can be sourced from wherever you can get it)
- Trypsin (Promega, CAT#V5073)
- Tandem Mass Tag Reagents (Pierce)

- Glycine (Sigma, CAT#G8898)
- Xylenes (Sigma, CAT#534056)
- cOmplete protease inhibitor cocktail, without EDTA (Roche, sold by Sigma, CAT#11873580001)

Pre-lysis Buffer

Reagent	Stock Concentration	Final Concentration
HEPES buffer, pH 8.5	200mM	50mM
SDS	20% (w/v)	1%

Day 1 Protocol (~4 hours)

1. If sections are FFPE and have not been deparaffinized, start here, otherwise skip to step 7.
2. Spin tubes with sections for 1 minute at 20,000g.
3. Add 800uL of xylene and vortex for 10 seconds.
4. Spin tubes for 1 minute at 20,000g and discard the xylene supernatent into a waste tube (in the fumehood!)
5. Add 800uL of ethanol and vortex for 10 seconds.
6. Spin tubes for 1 minute at 20,000g and discard the ethanol. Leave tubes open to air dry. Want to remove as much ethanol here as possible while still keeping the sections damp.
7. Add 2uL of benzonase to a PCR tube for each sample to be processed.

8. Add 40uL of pre-lysis buffer to each PCR and pipette mix.
9. Using a 200uL pipette tip, transfer the tissue material to the PCR tube and mix around to dissociate it a bit.
10. Incubate at 37C for 30 minutes.
11. Make enough SDS and DTT for the samples by mixing 25uL of DTT with 475uL of 20% SDS.
12. Add 40uL of the SDS-DTT mix to each tube and mix by pipetting. (TECHNIQUE: typically I will use the pipette tip to slide the intact tissue piece up the side of the tube a bit out of the liquid, mix the liquid, and then slide the tissue back in).
13. Incubate the tubes at 70C for 2 hours in a PCR thermocycler.
14. Turn on the Bioruptor Pico to begin chilling.
15. Just prior to step 14 completing, make a mixture of SP3 beads with IAA. Use 10uL of each SP3 bead stock per sample (e.g. for 10 samples, 100uL of each bead type). Rinse the beads 1X with 200uL of water, and reconstitute in 10uL of IAA per sample (e.g. 100uL for 10 samples).
16. Allow the samples to cool and add 10uL of the IAA SP3 bead mix to a fresh 0.5mL eppendorf tube.
17. Transfer the liquid and tissue chunks to the eppendorf tubes and mix.
18. Sonicate the tubes in the Bioruptor for 20 cycles (30 seconds ON, 30 seconds OFF). See the 'Bioruptor Pico Usage' protocol on this wiki for directions.
19. Let the samples sit in the dark at room temperature for 30 minutes after the sonication.

20. Transfer the liquid and tissue back to the same PCR tubes for SP3.
21. Thaw a tube of the eColi internal standard from the MS Standards box in the -20C. This is a standard eColi lysate that has been reduced and alkylated. We typically add 10ug of protein per 10-plex (1ug per channel).
22. Take 3.5uL of the eColi stock and dilute it to 50uL with water.
23. Add 5uL of this standard to each sample and pipette mix.
24. Add 70uL of acetonitrile to each tube (do not attempt to pipette mix, just shoot the liquid in). Mix the tubes by inverting them.
25. Incubate the tubes for 8 minutes at room temperature.
26. Place on a magnetic rack, and rinse 2X with 70% ethanol, and 1X with acetonitrile - while on the rack! NOTE: the tissue that is remaining should remain bound to the beads through this entire process.
27. Prepare trypsin by reconstituting 20ug of trypsin solid in 200uL of 0.2M HEPES pH 8.
28. Add 5uL of this trypsin to the bottom of each sample tube.
29. Add a further 50uL of 0.2M HEPES pH 8 to each tube, pipette mix the trypsin in, and gently push the bead and tissue chunks into the liquid. Do not attempt to pipette mix them.
30. Sonicate the tubes in a water bath for a few seconds until the beads look dispersed.
31. Digest overnight at 37C (14 hours minimum).

Day 2 Protocol (~1 hour)

1. Sonicate the tubes in a water bath for a few seconds to reconstitute the beads.
2. Add 50uL of ACN to each tube and briefly pipette mix.
3. Incubate for 8 minutes at room temperature.
4. Place the tubes on a magnetic rack. Recover the liquid to 1.5mL tubes.
5. Transfer 10uL of each sample to a fresh tube to create your 'pooled' sample.
6. Transfer 10uL of each of these tubes to a new 1.5mL tube containing 100uL of H2O + 0.1% formic acid and pipette mix. These samples can be Stage Tipped and injected as test samples to test the quality and amount of sample. Wait to see the result of the test samples before proceeding with the TMT labeling. Samples can be stored at -20C.

Day 3 Protocol (~1 hour)

1. Thaw the TMT 10plex reagents and let settle to room temperature for 10 minutes. Briefly mix the tubes by shaking (reagents are at a stock concentration of 10ug/uL).
2. Adjust the amount of volume of each sample used based on the results of the test injections. Bring the total volume to 90uL of each sample using 0.2M HEPES pH 8 prior to TMT labeling.
3. Add 8uL of the appropriate TMT reagent to each tube, pipette mix, and incubate for 30 minutes at room temperature. In a standard case you have 9 samples (TMT1-9), the pooled sample (TMT10).

4. Add a further 8uL of the appropriate TMT reagent to each tube, pipette mix, and incubate for 30 minutes at room temperature.
5. Add 10uL of 1M glycine to each tube and pipette mix. Incubate for 10 minutes at room temperature.
6. Combine the liquid from the 10 tubes into a single fresh 1.5mL tube.
7. SpeedVac the combined tube to dryness, or until the volume is <100uL.
8. Reconstitute the dried sample with 0.1% formic acid in water until the final volume is 600uL.
9. Desalt the sample using the SepPak protocol found on this website.
10. SpeedVac the eluted sample to dryness or until the volume is <100uL.
11. Bring the sample to at least 50uL with water if it went below this volume during the SpeedVac process. Vortex the tube briefly.
12. Add 30uL of 1M NaOH to a fresh 1.5mL tube.
13. Spin the 50uL SepPak'd sample at 20,000g for 2 min, transfer the sample to the tube with 30uL NaOH and pipette mix.
14. Add 50 uL of 100mM borate buffer, pH 9.3 (mobile phase A for fractionation).
15. Add H2O to make a total final volume of 200uL.
16. Ensure that the pH is basic using pH paper. If it is not, make it basic by adding more 1M NaOH.
17. Spin the sample at 20000g for 5 minutes, and collect the supernatant in a fresh 1.5mL tube.
18. Store the sample at -20C until HPLC fractionation.

19. Proceed to High-pH Reversed Phase fractionation with 50% of your sample. Use the 50% for strong anion exchange fractionation.

Prepare for MS analysis

1. Reconstitute each of your fractions in 20uL of 1% formic acid.
2. Add 8uL of this sample to the MS well plate, and inject 3uL to the MS.

Supplemental References

1. Hughes, C. S. *et al.* Ultrasensitive proteome analysis using paramagnetic bead technology. *Mol. Syst. Biol.* **10,** 1–14 (2014).
2. Anglesio, M. S. *et al.* Type-Specific Cell Line Models for Type-Specific Ovarian Cancer Research. *PLoS One* **8,** (2013).
3. Rappsilber, J., Ishihama, Y. & Mann, M. Stop and go extraction tips for matrix-assisted laser desorption/ionization, nanoelectrospray, and LC/MS sample pretreatment in proteomics. *Anal. Chem.* **75,** 663–70 (2003).
4. Yang, F. & Shen, Y. High-pH reversed-phase chromatography with fraction concatenation for 2D proteomic analysis. *E Rev. Proteomics* **9,** 129–134 (2012).
5. Uhlen, M. *et al.* Tissue-based map of the human proteome. *Science (80-.).* **347,** 1260419–1260419 (2015).
6. Yamaguchi, K. *et al.* Identification of an ovarian clear cell carcinoma gene signature that reflects inherent disease biology and the carcinogenic processes. *Oncogene* **29,** 1741–1752 (2010).
7. Uehara, Y. *et al.* Integrated Copy Number and Expression Analysis Identifies Profiles of Whole-Arm Chromosomal Alterations and Subgroups with Favorable Outcome in Ovarian Clear Cell Carcinomas. *PLoS One* **10,** (2015).
8. TCGA. Integrated genomic analyses of ovarian carcinoma. *Nature* **474,** 609–615 (2011).
9. Zhang, H. *et al.* Integrated Proteogenomic Characterization of Human High-Grade Serous Ovarian Cancer. *Cell* (2016). doi:10.1016/j.cell.2016.05.069
10. Tripathi, S. *et al.* Meta- and Orthogonal Integration of Influenza ‘OMICs’ Data Defines a Role for UBR4 in Virus Budding. *Cell Host Microbe* **18,** 723–35 (2015).

Supplemental Figure Legends

Supplemental Fig. 1 – SP3-CTP enables robust proteome analysis of FFPE tissue sections. Sets of 10 unique tumour samples (5 HGSC, 5 CCC) were prepared in biological duplicate (serial sections) for proteomics analysis using SP3-CTP. Samples were analyzed in two separate 10-plex TMT experiments on an Orbitrap Fusion MS with MS3 scanning. **(a)** The comprehensiveness of the proteomic data is illustrated with a histogram coverage map of the FFPE data with RNA-seq expression values derived from normal ovarian tissue sample from the Human Protein Atlas [5]. The numbers in the insets indicate the number of unique genes identified within the given ranges (>1 FPKM or <1 FPKM). A total of 313 proteins identified had no matching RNA-seq read. **(b)** The density of the identification matrix is illustrated by a cumulative Venn diagram showing the number of new unique proteins added to the total dataset by including additional tumours from the 10-plex set. The analysis starts with the tumour containing the smallest number of missing proteins, and adds tumours sequentially based on increasing numbers of missing values. The numbers beside each patient indicate the number of proteins found in that sample alone. **(c)** The density of the quantification matrix is displayed using a heat map of individual tumour samples with proteins denoted by colored boxes. The red dashed line indicates the divide between single and multi-peptide protein hits. The inset heat map displays single peptide hits that represent the majority of proteins with missing values.

Supplemental Fig. 2 – Sample processing and peptide quantification are highly reproducible when using the SP3-CTP workflow. Sets of 10 unique

tumour samples (5 HGSC, 5 CCC) were prepared for proteomics analysis using SP3-CTP. The set of 10 tumours was prepared in biological duplicate (serial sections processed individually, e.g. Set A1 vs. B1) and analyzed in technical duplicate (multiple injections of the same fractions, e.g. Set A1 vs. A2). Each TMT channel was spiked with a lysate derived from *E. coli* prior to digestion to monitor processing reproducibility. **(a)** Smoothed density scatter plot depicting the reproducibility between technical replicates. Values in the inset indicate Pearson correlation values of peptide expression between technical replicates belonging to each biological replicate. **(b)** Smoothed density scatter plot depicting reproducibility between biological replicate samples. Technical replicates were averaged for each biological duplicate set prior to plotting. **(c)** Relative log2 expression of *E. coli* peptides across all biological and technical replicates for each of the 10-plex batches. All values are relative to the median value per peptide. Centre lines in plotted boxes indicate the median, upper and lower the 75th and 25th percentiles, and upper and lower whiskers 1.5X the interquartile range. **(d)** Density smoother scatter plot of fold change values derived from the comparison of HGSC with CCC from the FFPE tumour samples for the two biological replicates. All correlation values are calculated using the Pearson method.

Supplemental Fig. 3 – Identification and quantification metrics of the FFPE tumour data derived from SP3-CTP are of high quality. **(a)** Histogram plot of the number of unique peptides identified per protein in the FFPE data combining all technical and biological replicates. **(b)** Histogram of the counts of total proteins

identified as having a significant change in expression between HGSC and CCC based on the number of unique peptides found for each. Inset table displays the numbers of proteins found in the low peptide number bins.

Supplemental Fig. 4 – Identification and expression metrics from FFPE tissues correlate highly with matched frozen specimens. Sets of 8 unique tumour samples (4 HGSC, 4 CCC) were prepared in biological duplicate (serial sections) for proteomic analysis using SP3-CTP. Samples were analyzed in two separate 10-plex TMT experiments on an Orbitrap Fusion MS with MS3 scanning. Each of the frozen tumour samples was from a matched individual in the previous FFPE set. **(a)** The comprehensiveness of the proteomic data is illustrated with a histogram coverage map of the frozen data with RNA-seq expression values derived from a normal ovarian tissue within the Human Protein Atlas. The numbers in the insets indicate the number of unique genes identified within the given ranges (>1 FPKM or <1 FPKM). **(b)** Density smoothed scatter plot of fold change values derived from the comparison of HGSC with CCC from the frozen tumour samples for the two biological replicates. All correlation values are calculated using the Pearson method.

Supplemental Fig. 5 – Deep and reproducible proteome coverage obtained in cell line samples with SP3. A set of 6 individual cell line samples (3 HGSC, 3 CCC) were analyzed in biological duplicate for proteomics using a modified SP3-CTP protocol with TMT 6-plex labeling on an Orbitrap Fusion MS with MS3 scanning. **(a)** Schematic of the analysis pipeline used to process the cell line samples with an SP3-based pipeline. **(b)** The comprehensiveness of the

proteomic data is illustrated with a histogram coverage map of the cell line data with RNA-seq expression values derived from a normal ovarian tissue within the Human Protein Atlas. The numbers in the insets indicate the number of unique genes identified within the given ranges (>1 FPKM or <1 FPKM). **(c)** Density smoothed scatter plot of fold change values derived from the comparison of HGSC with CCC from the cell line samples for the two biological replicates. All correlation values are calculated using the Pearson method.

Supplemental Fig. 6 – Comprehensive proteome analysis of ovarian carcinoma histotypes using an SP3-CTP pipeline. Sets of unique tumours (6 HGSC, 6 CCC, 6 ENOC) were prepared and analyzed using the SP3-CTP workflow. Samples were processed in two batches (3 HGSC, 3 CCC, 3 ENOC) in biological triplicate (3 serial 10µm sections). A pooled internal standard containing an aliquot of all 54 samples was used as the 10th TMT channel. A standard mix of an *E. coli* lysate was spiked into each sample to monitor processing reproducibility. **(a)** Schematic of the processing and data analysis workflow used for SP3-CTP. **(b)** The comprehensiveness of the proteomic data is illustrated with a histogram coverage map of the 18-tumour proteomics data with RNA-seq expression values derived from a normal ovarian tissue within the Human Protein Atlas. The numbers in the insets indicate the number of unique genes identified within the given ranges (>1 FPKM or <1 FPKM). **(c)** Relative log2 expression of *E. coli* peptides across all biological and technical replicates for each of the 10-plex batches. All values are relative to the median value per peptide. Centre lines in plotted boxes indicate the median, upper and lower the

75th and 25th percentiles, and upper and lower whiskers 1.5X the interquartile range.

Supplemental Fig. 7 – Comparing CCC molecular signature with the expression variance observed between histotypes. The CCC signature captured from gene expression data [6] was compared with the 18-tumour proteomics data set to measure the expression patterns of these features. **(a)** Volcano plot of expression between the HGSC and CCC histotypes with proteins detected in SP3-CTP from the signature gene set (n=183) overlaid and highlighted. Blue points indicate genes that are upregulated in CCC, and red those that are down based on gene expression. PECA score represents the median adjusted p-value of all peptides assigned per protein. Genes listed in the inset boxes are the top 10 differentially expressed based on adjusted p-values. The dotted vertical dashed lines indicate one standard deviation from the mean fold change. **(b)** Clustering of the 18-tumour proteomics data using the set of features that were reliably identified across all histotypes (n=75) [6]. Tumour content estimates are based on histological examination of tissue sections (see Methods). **(c)** Clustering of the 18-tumour proteomics data using the set of features that were reliably identified across all histotypes (n=75). Values in cells represent the Pearson correlation values between the listed samples.

Supplemental Fig. 8 – RNA and protein expression values from FFPE tissue samples display significant correlation. The set of 54 individual samples (tumours + replicates) was compared to determine differential protein expression between histotypes. Each data set was compared with RNA expression values

derived from microarray analysis of ovarian tumours [7]. The correlation of protein and RNA fold change values between HGSC and CCC with the protein features identified in the 113-gene set highlighted for **(a)** 18-tumour set, **(b)** FFPE, **(c)** frozen, **(d)** cell line sample types. All correlated values are calculated using the Pearson method. Marker correlations include only the highlighted feature genes.

Supplemental Fig. 9 – Differential protein expression analysis reveals histotype diversity in ovarian carcinomas. The set of 54 individual samples from the 18-tumour set (tumours + replicates) was compared to determine differential protein expression between histotypes. Pre-classified tumours belonging to a single histotype were treated as replicates for statistical analyses. Volcano plots depicting the variance in expression between **(a)** HGSC and ENOC and **(b)** CCC and ENOC histotypes. Colored points indicate those exceeding an adjusted p-value threshold of 0.05. PECA score represents the median adjusted p-value of all peptides assigned per protein. Genes listed in the inset boxes are the top 10 differentially expressed based on adjusted p-values. The dotted vertical dashed lines indicate one standard deviation from the mean fold change. The correlation between RNA expression values derived from microarray experiments can be observed for **(c)** HGSC vs. ENOC and **(d)** CCC vs. ENOC histotypes. All correlation values are calculated using the Pearson method.

Supplemental Fig. 10 – Extracting features of ovarian carcinoma histotypes using protein expression values determined using SP3-CTP. To capture protein features that are characteristic of the ovarian carcinoma histotypes,

expression values from HGSC, CCC, and ENOC were compared. Scatter plot of comparisons between HGSC, CCC, and ENOC. Coloured markers are taken from proteins identified in the 113-gene set. Protein features that displayed reproducible expression that were selected for further investigation are highlighted with open black circles. Correlation between the two data sets was calculated using the Pearson method.

Supplemental Fig. 11 – Examination of HGSC features with high and low expression across cancer types found in the TCGA. To monitor expression across cancers, the top high and low HGSC SP3-CTP features (MSLN, LEFTY1) were examined across the displayed tumour types using TCGA gene expression data **(a-b)**. Overlaid points are the values assigned to each tumour. Centre lines in plotted boxes indicate the median, upper and lower the 75th and 25th percentiles, and upper and lower whiskers 1.5X the interquartile range. **(c)** For methylation analysis, a set of the top 500 genes that explain the majority of the variance in the proteomics data based on PCA (Figure 4a) were extracted from the 18-tumour proteomics data. Analysis of promoter methylation was queried using cBioPortal for values derived from the in-depth analysis of HGSC ovarian carcinoma by the TCGA. Relative log2 expression values are calculated as the mean difference of HGSC tumours relative to the median across all tumours per gene. **(d)** Expression dynamics of markers of poor or good prognosis from the TCGA gene expression data was performed using the total set of identified proteins from the 18-tumour proteomics data. Statistical analysis and classification of prognosis were performed as described previously [8].

Supplemental Fig. 12 – Examination of HGSC features with high and low expression across cancers in the Human Protein Atlas. To monitor expression across cancers, the top high and low HGSC features (MSLN, LEFTY1) were examined across the displayed types using data taken from the gene expression data in the Human Protein Atlas **(a-b)**. Numerical expression values are calculated based on assigning a score of 9 for 'High', 6 for 'Medium', 3 for 'Low', and 0 for 'Not detected'. Each score is multiplied by the number of assigned tumours for each category, and summed across each gene to give a single numerical estimate of expression per protein.

Supplemental Fig. 13 – Examination of HGSC features with high and low expression across the CPTAC analysis of ovarian carcinoma. From the set of 18 unique tumours analysed, histotypes were cross-compared to generate sets of enriched features for each. **(a)** Expression of HGSC features in MS-based proteomics data taken from the CPTAC study of ovarian carcinoma [9]. Values are log2 ratios relative to the reference standard used in each experimental batch. Only 'unshared' log2 ratios were used in this analysis. Data were from a combined set of data analysed at two separate institutes (Supplemental Table 5). Centre lines in plotted boxes indicate the median, upper and lower the 75th and 25th percentiles, and upper and lower whiskers 1.5X the interquartile range. Overlaid points represent values per individual. **(b)** The top 50 'High' and 'Low' expressed features were taken from the CPTAC ovarian carcinoma data based on the median of the sum of spectral counts across all pools at each institute.

Features were overlaid on the HGSC vs. CCC expression map from the 18-tumour SP3-CTP proteomics analysis. Marked genes denote those that exceed the score threshold in the expected fold-change direction. PECA score represents the median adjusted p-value of all peptides assigned to a protein.

Supplemental Fig. 14 – Examination of clear cell carcinoma features using protein expression measurements in cell lines. The protein features CTH and LEFTY1 were assayed in a panel of ovarian carcinoma cell lines using western blotting. **(a)** Processed western blot showing the staining of CTH and LEFTY1 in ovarian carcinoma cell lines of validated histotypes, with actin used for normalization purposes. **(b)** Raw western blot images for CTH, LEFTY1, and Actin from where the processed images were obtained.

Supplemental Tables

Supplemental Table 1 – Meta data for all tumour samples analyzed. Tumour sample information for the different batches of samples tested in this work. Cellularity is a measure of the area of the tissue block that is covered by cells. In the case of ovarian cancer, these are also used as tumour content measurements, as it is typical for the entire cellular content to be tumour cells.

Supplemental Table 2 – Variance in the 113-gene signature across data sets. A set of 113-genes that differentiates HGSC and CCC were built from multiple literature sources. Values in the columns are log2 fold change of HGSC relative to CCC for all of the different sample types examined in this study.

Supplemental Table 3 – Gene enrichment analysis in Metascape. All annotations and analyses are derived using Metascape. Gene identifiers were searched against GO and the HALLMARK (MSigDB) gene sets using Metascape. Derivation of enrichment scores was performed as described previously [10]. The InTerm_InList column denotes the numbers of genes present in the data query vs. those in the total list for that term. The Symbols column displays the genes that have mapped to that specific term.

Supplemental Table 4 – Proteomics mappings from MSigDB. All annotations are derived from the MSigDB resource. Gene sets are classified by the MSigDB collection, and the histotype that the expression values are derived from.

Supplemental Table 5 – Table of TCGA data sets used in this work. All data were accessed through cBioPortal using the R access point.

Supplemental Table 6 – Table of descriptions of R data storage objects containing data analysis tables. All data storage objects are available with the online material and can be accessed using the code provided in the Supplemental Protocols.

Supplemental Table 7 – Table of descriptions of files containing R code to perform all analyses used. All data processing files are openly accessible on GitHub (https://github.com/chrishuges/OvC), as well as with the online materials in ProteomeXChange.

Supplemental Table 8 – Table of descriptions of naming conventions in files uploaded to ProteomeXChange. All data described can be downloaded from the ProteomeXChange repository with the dataset identifier PXD003607.

Supplemental Figure 1

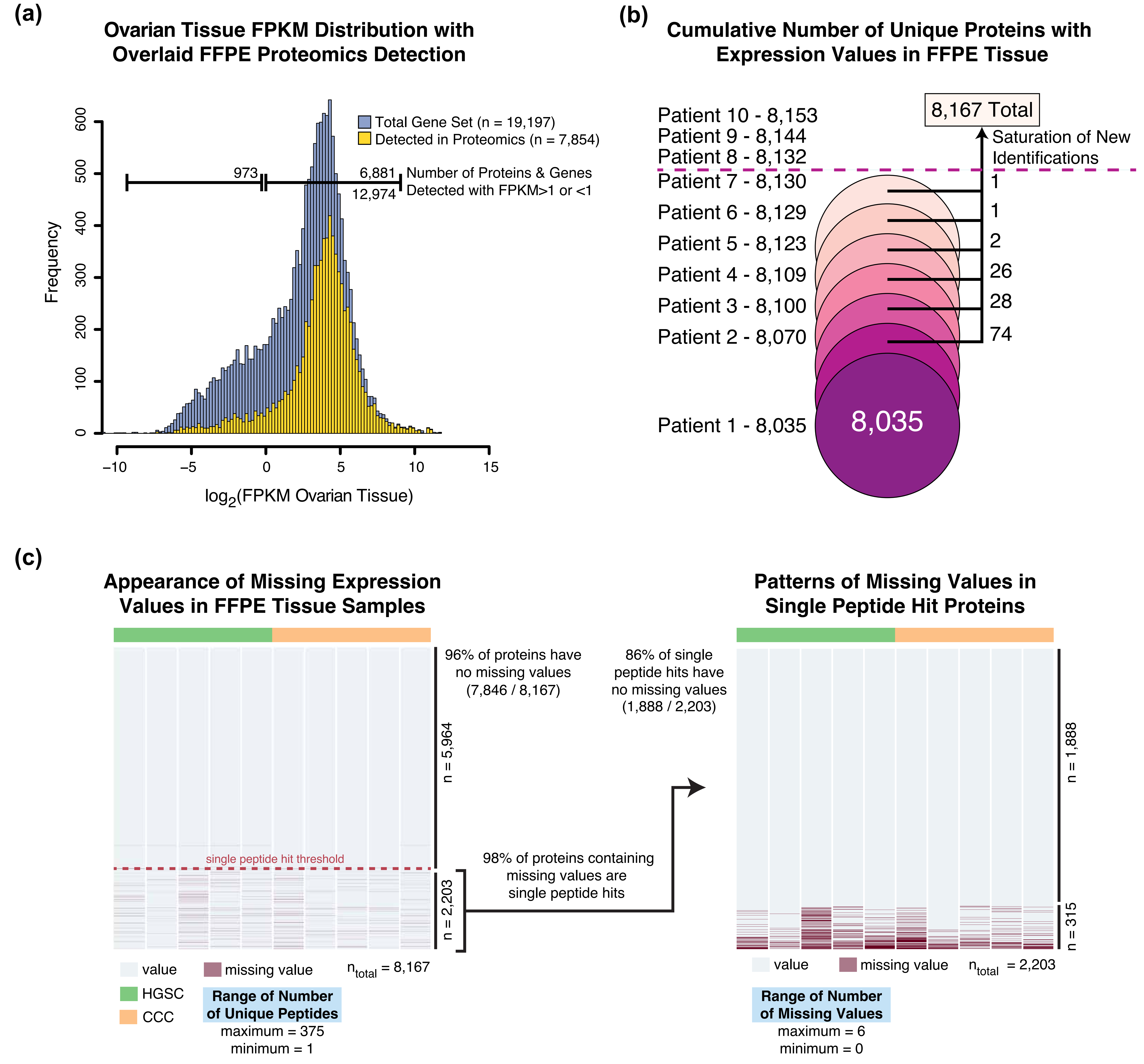

Supplemental Figure 2

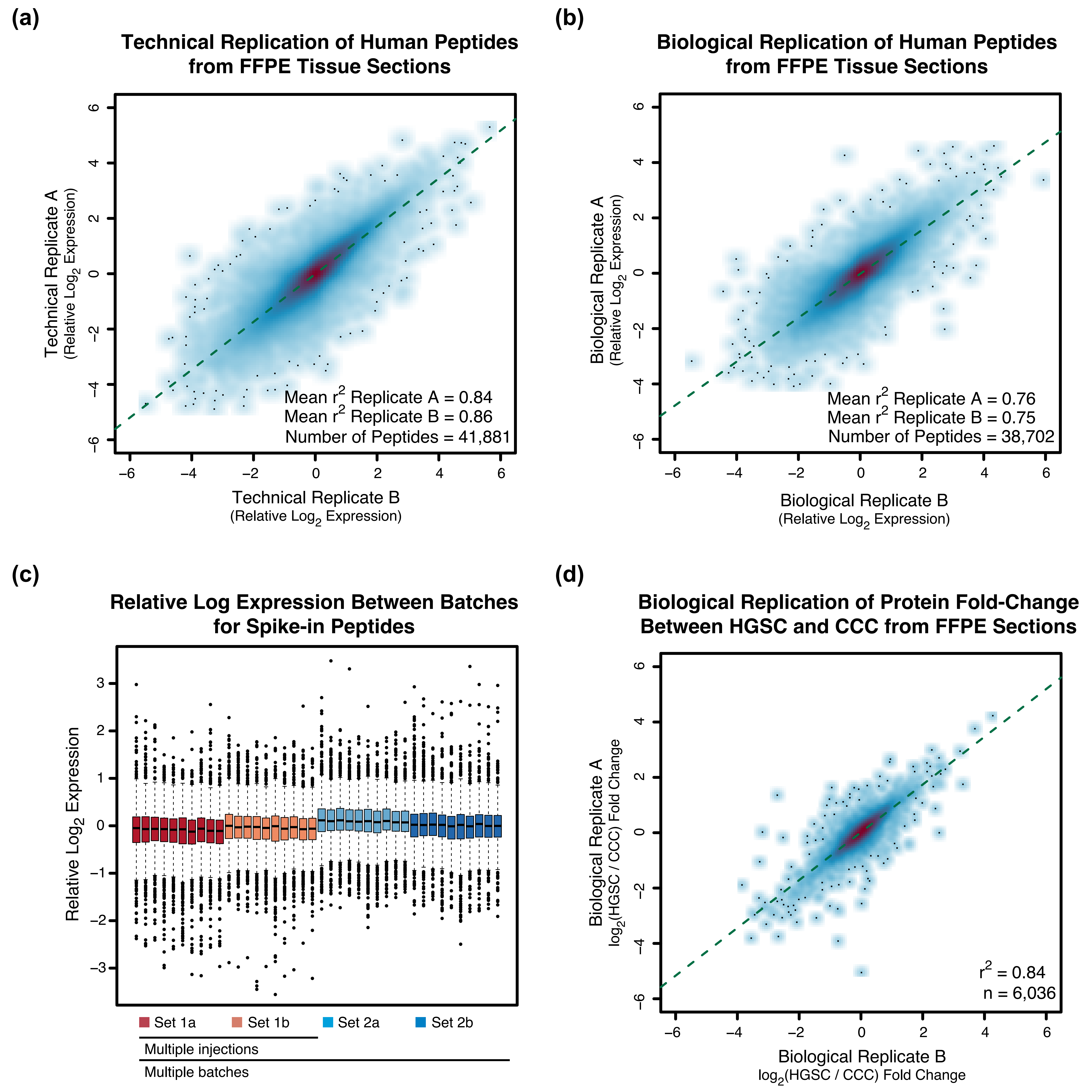

Supplemental Figure 3

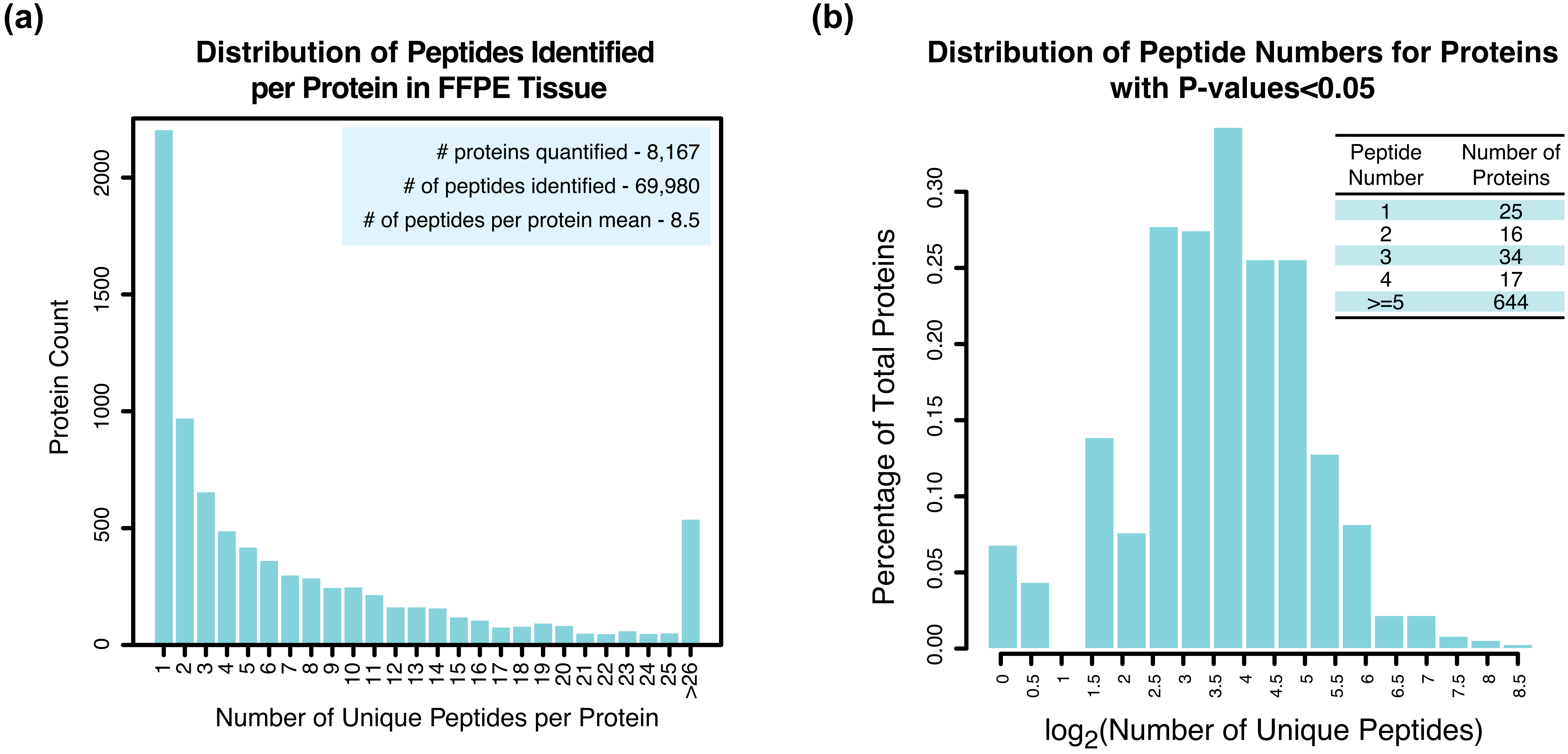

Supplemental Figure 4

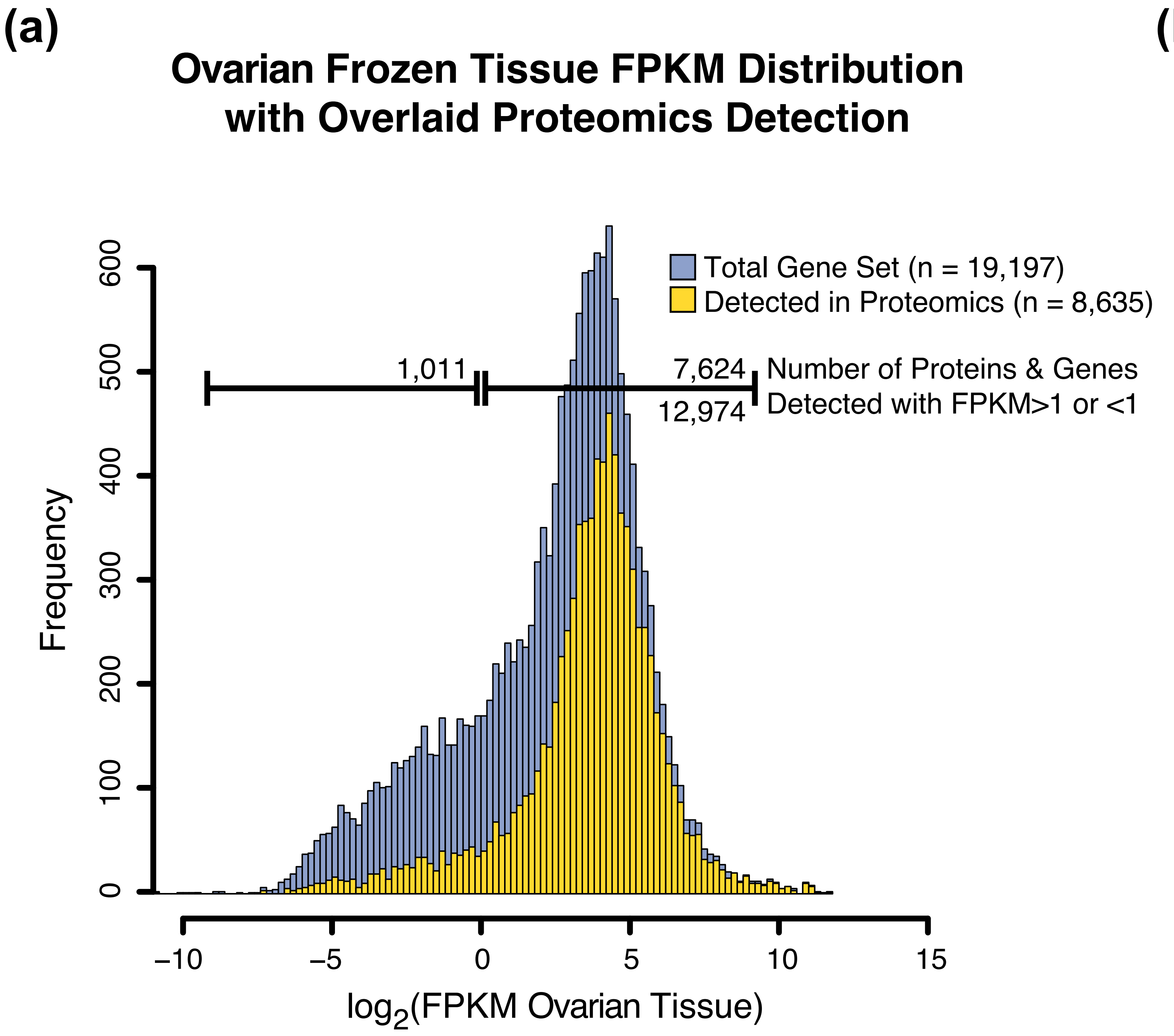

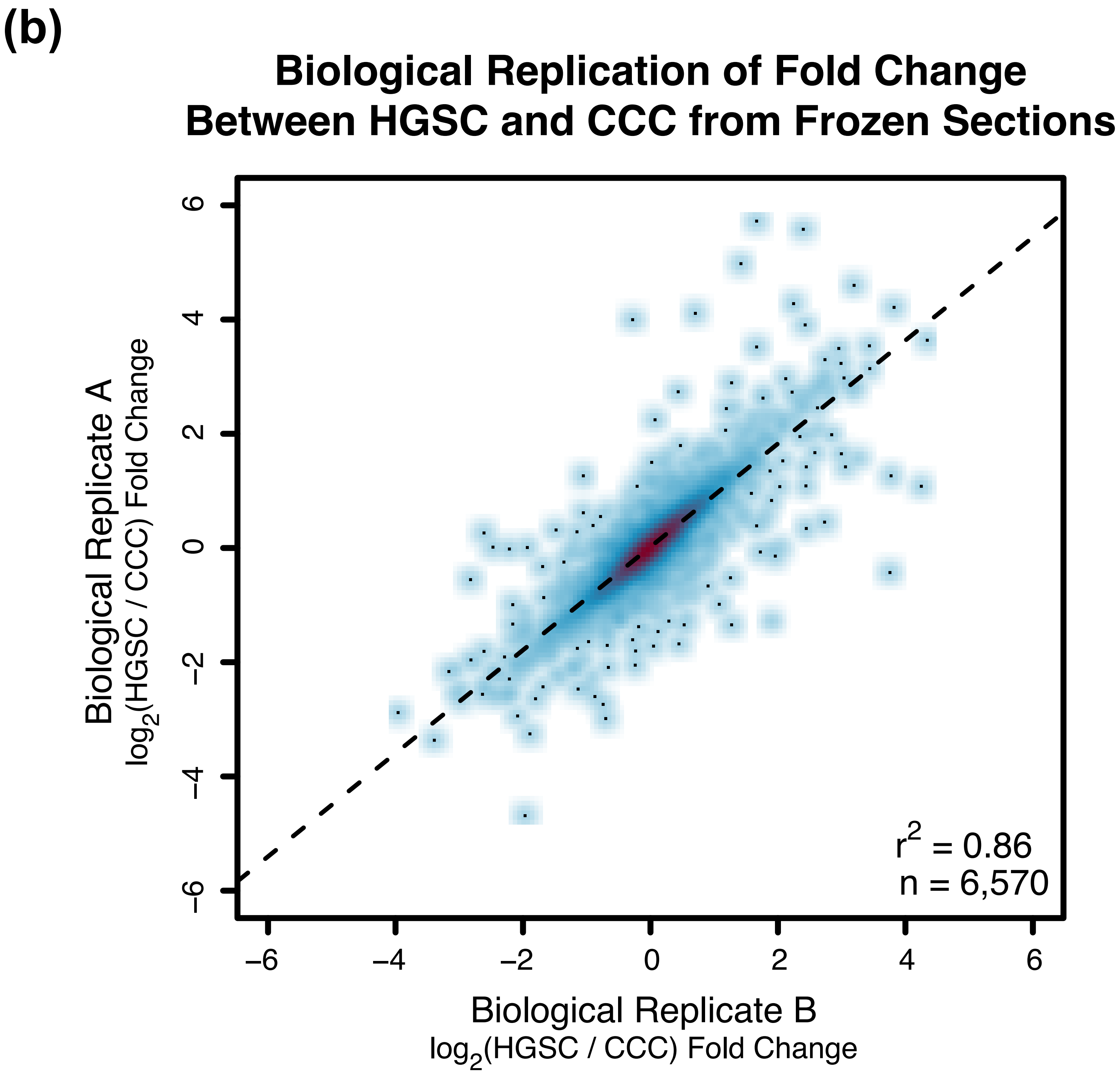

Supplemental Figure 5

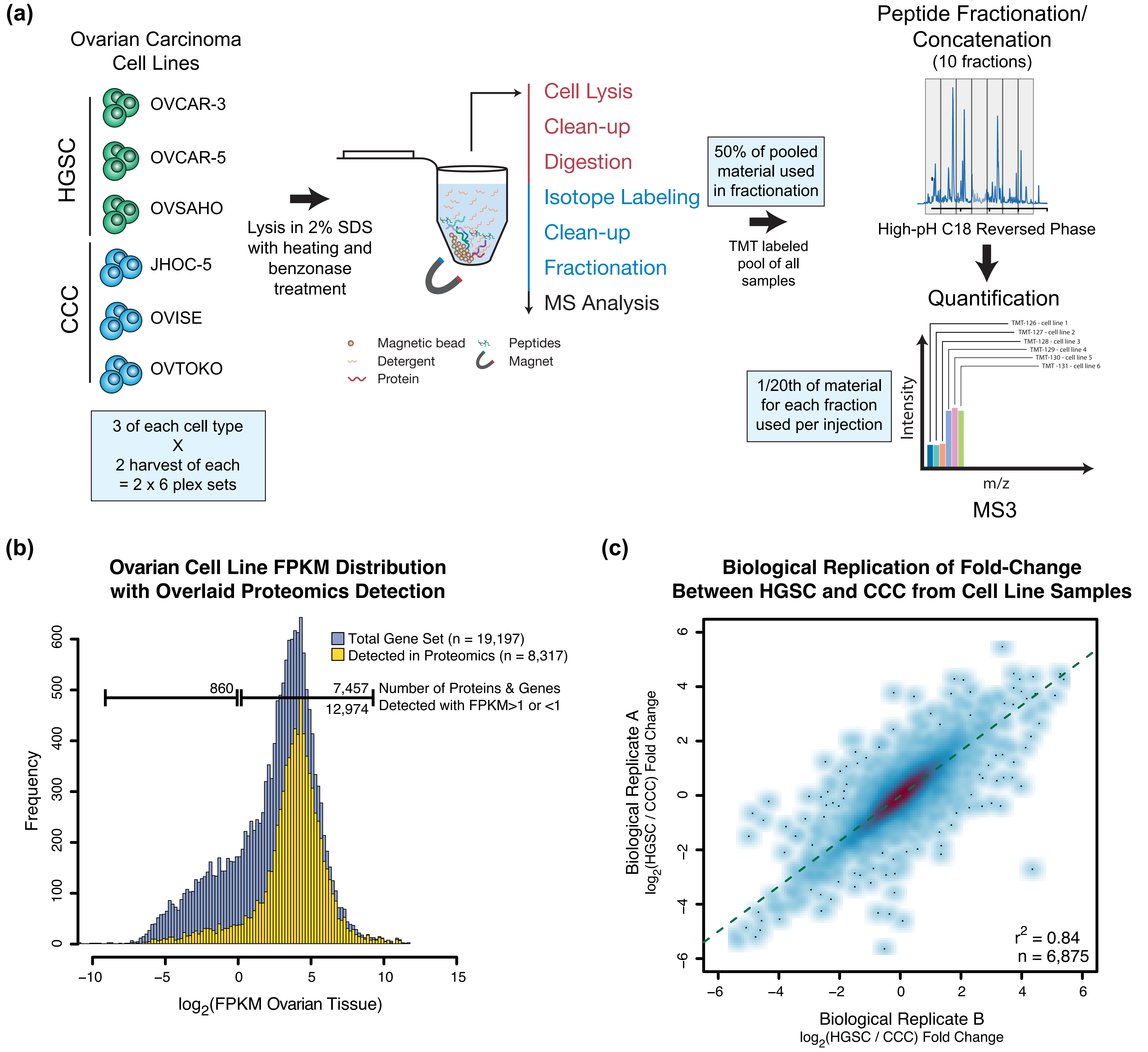

Supplemental Figure 6

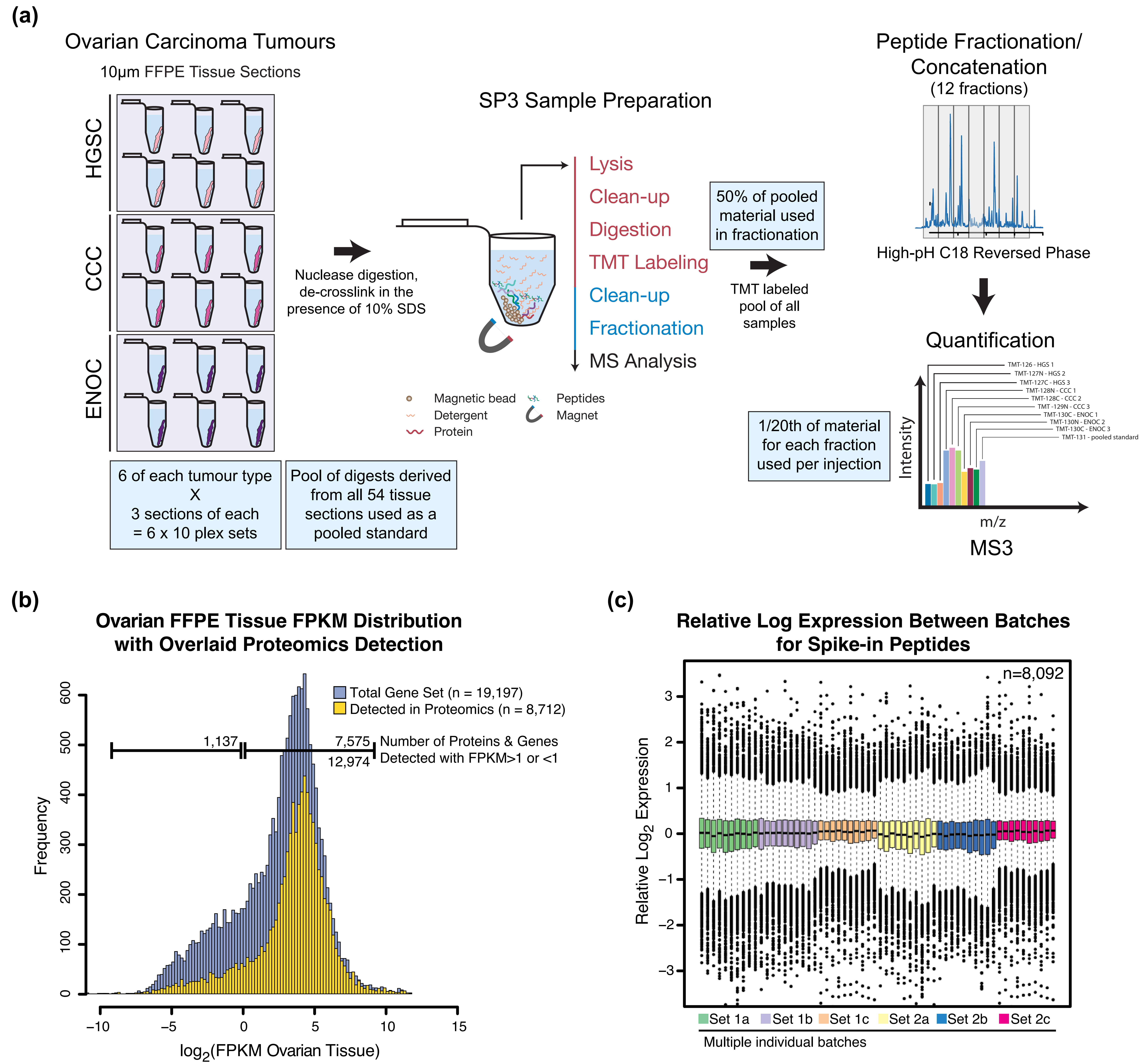

Supplemental Figure 7

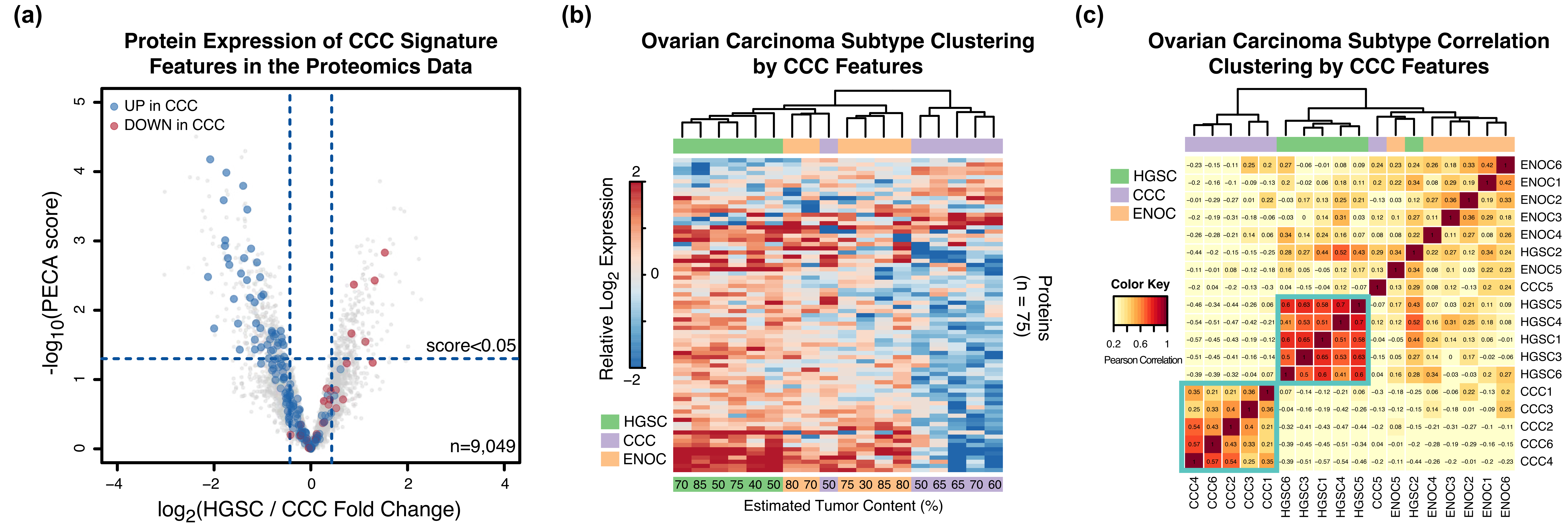

Supplemental Figure 8

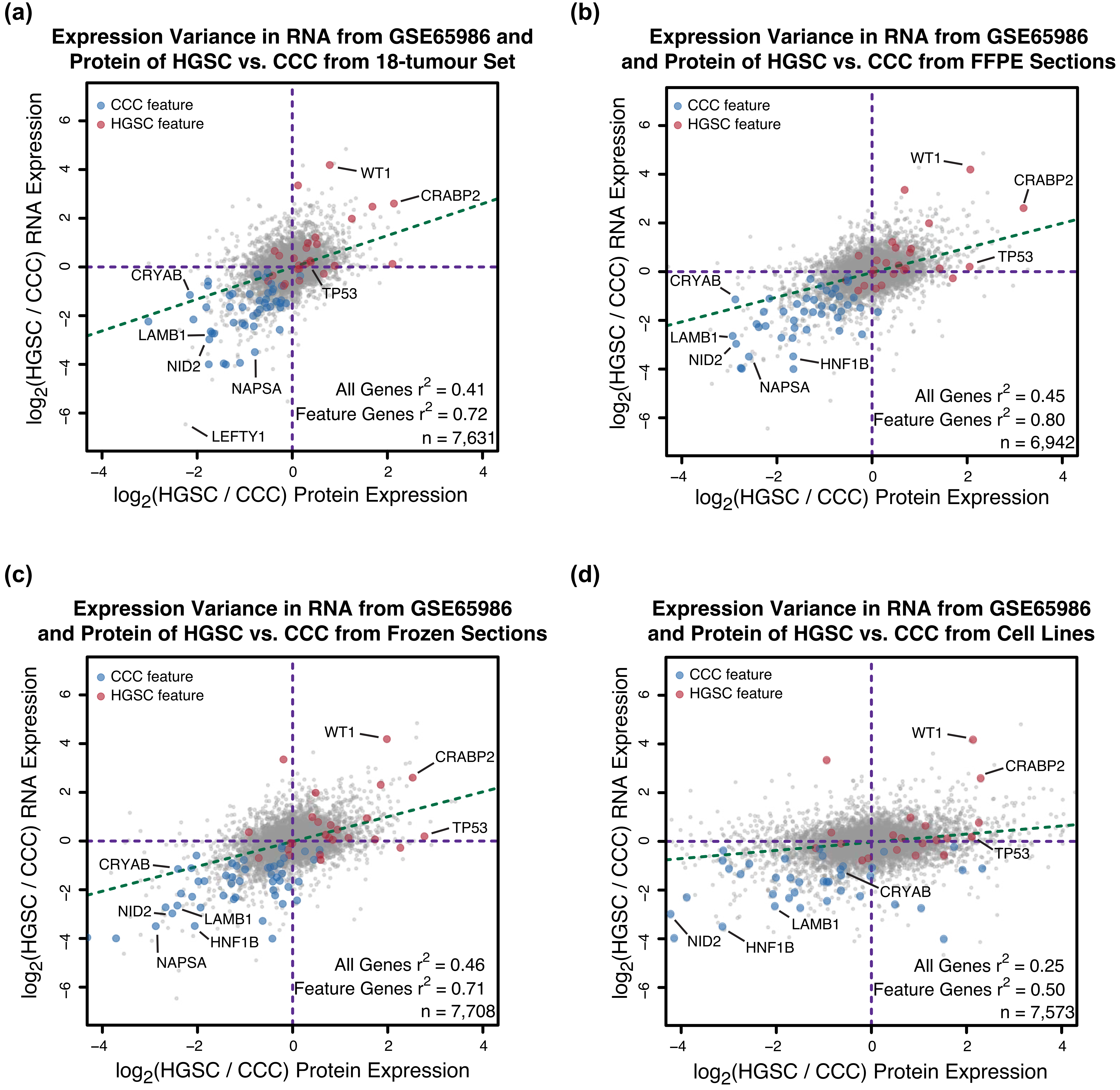

Supplemental Figure 9

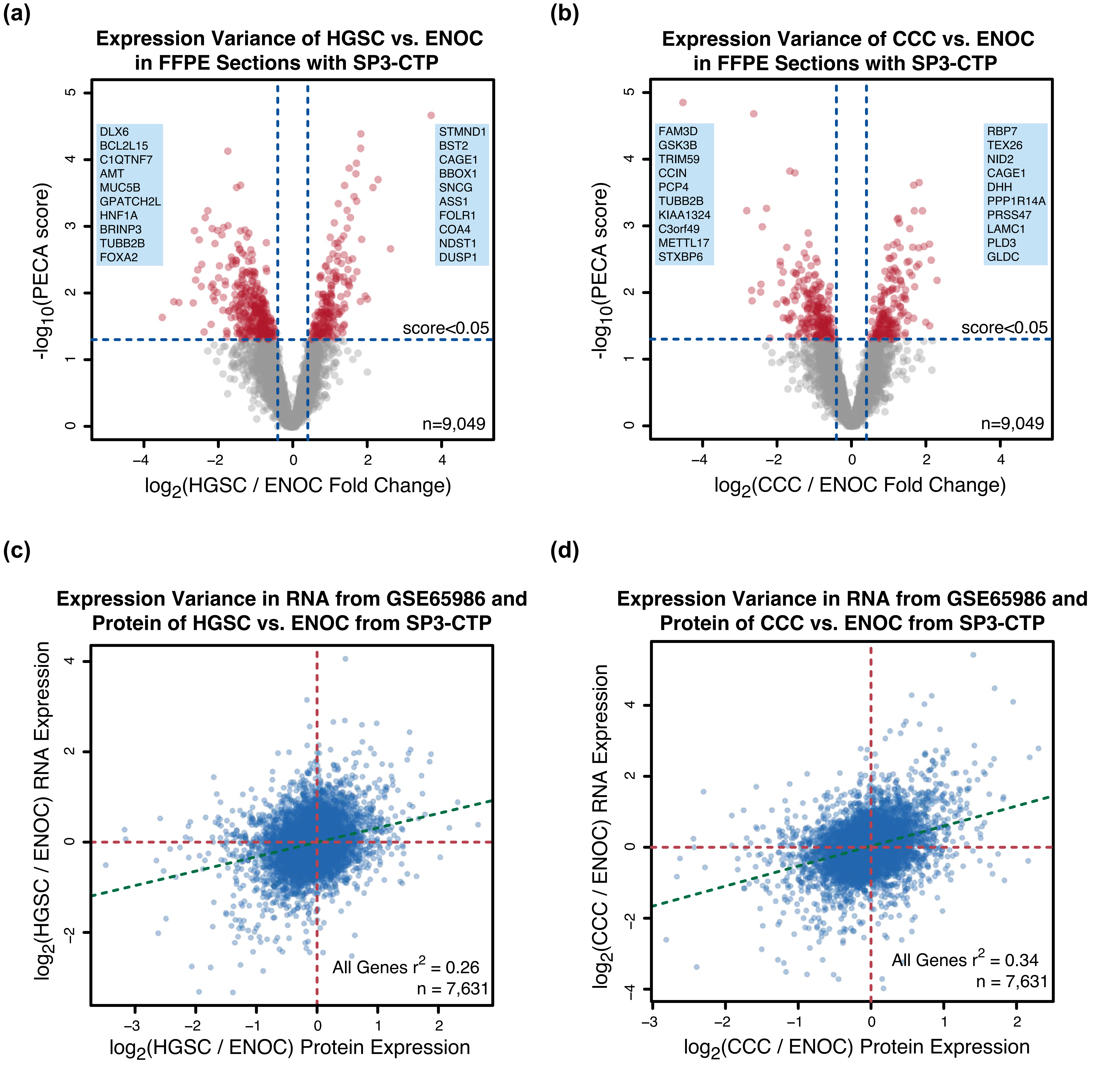

Supplemental Figure 10

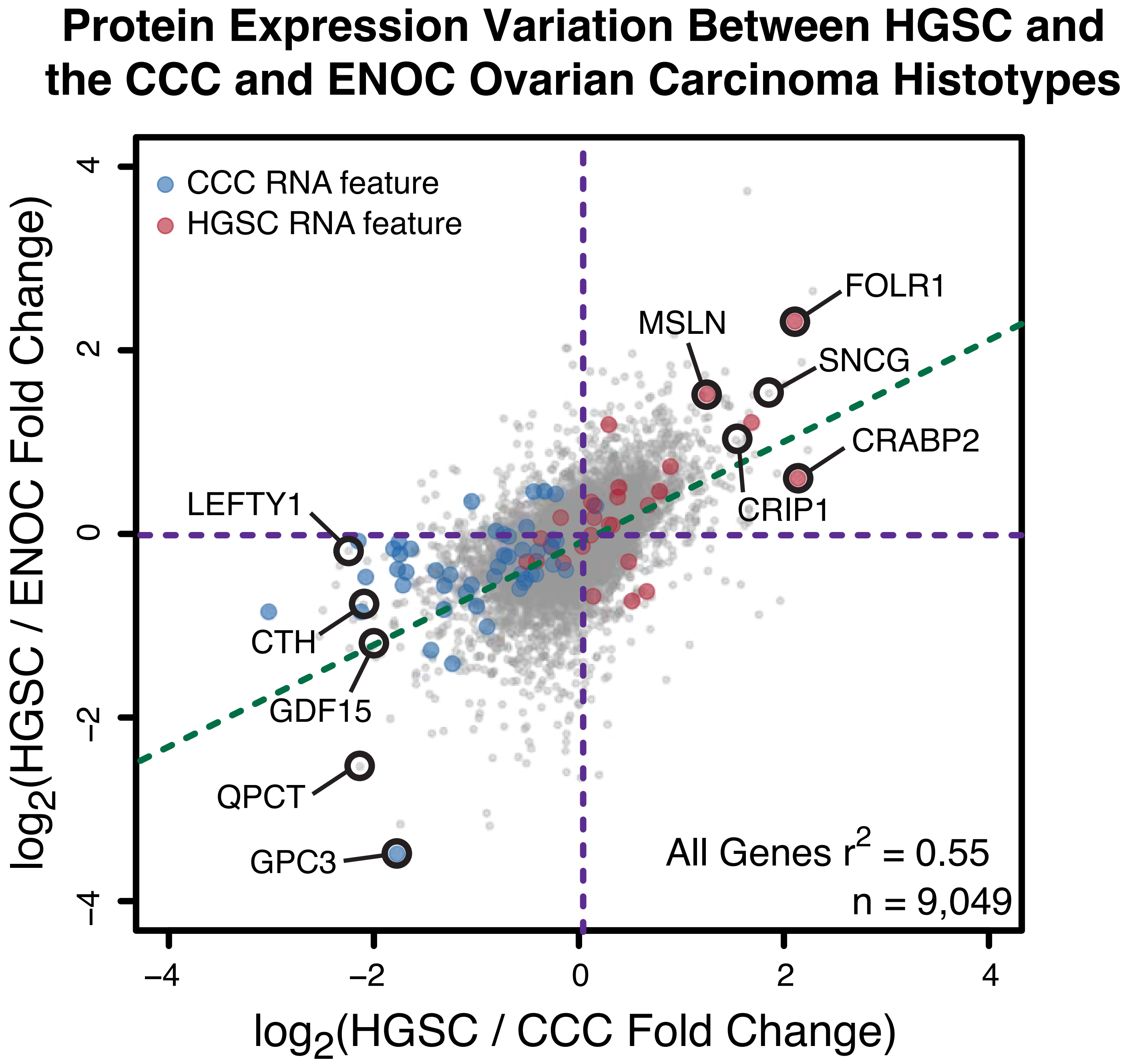

Supplemental Figure 11

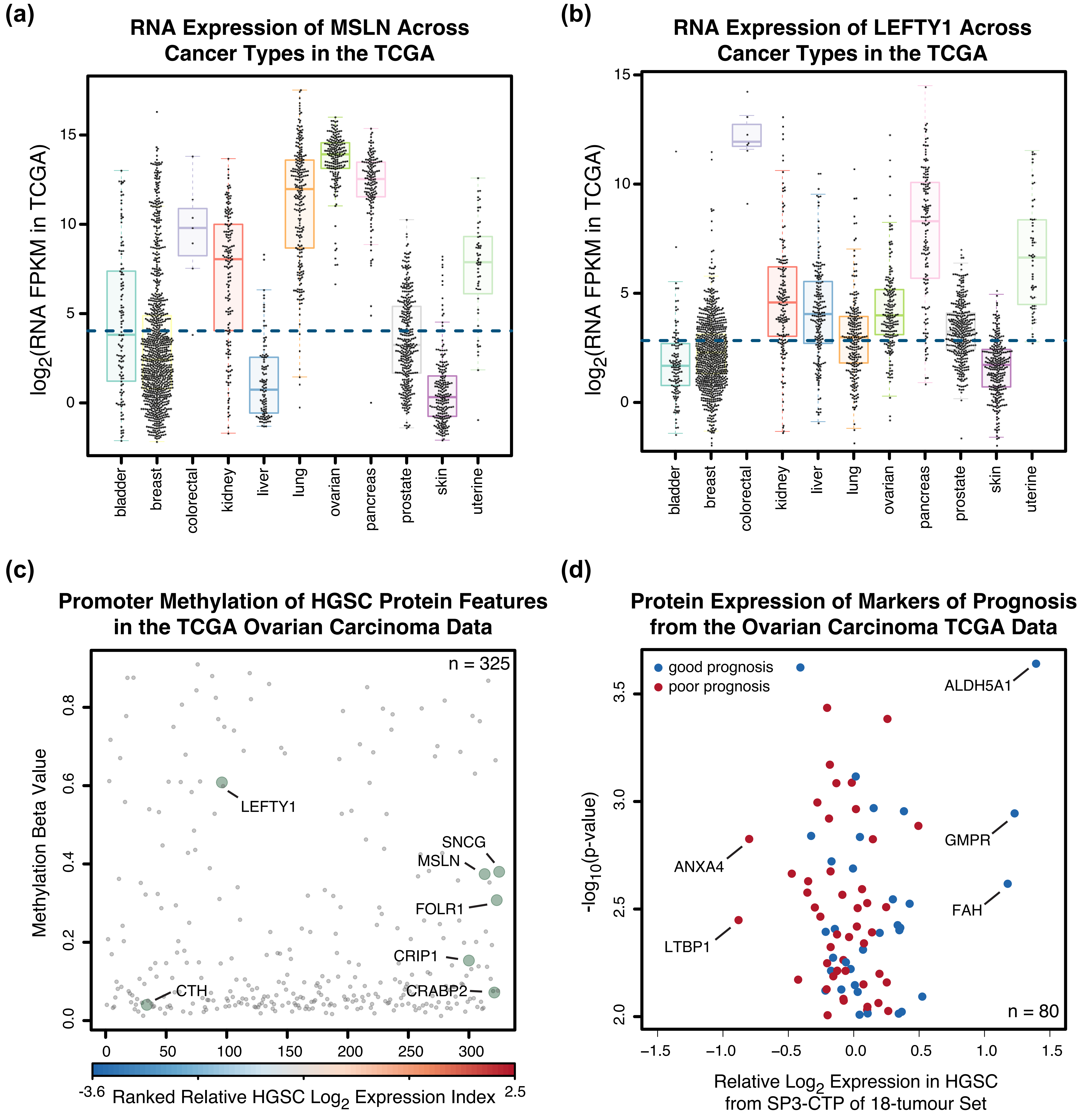

Supplemental Figure 12

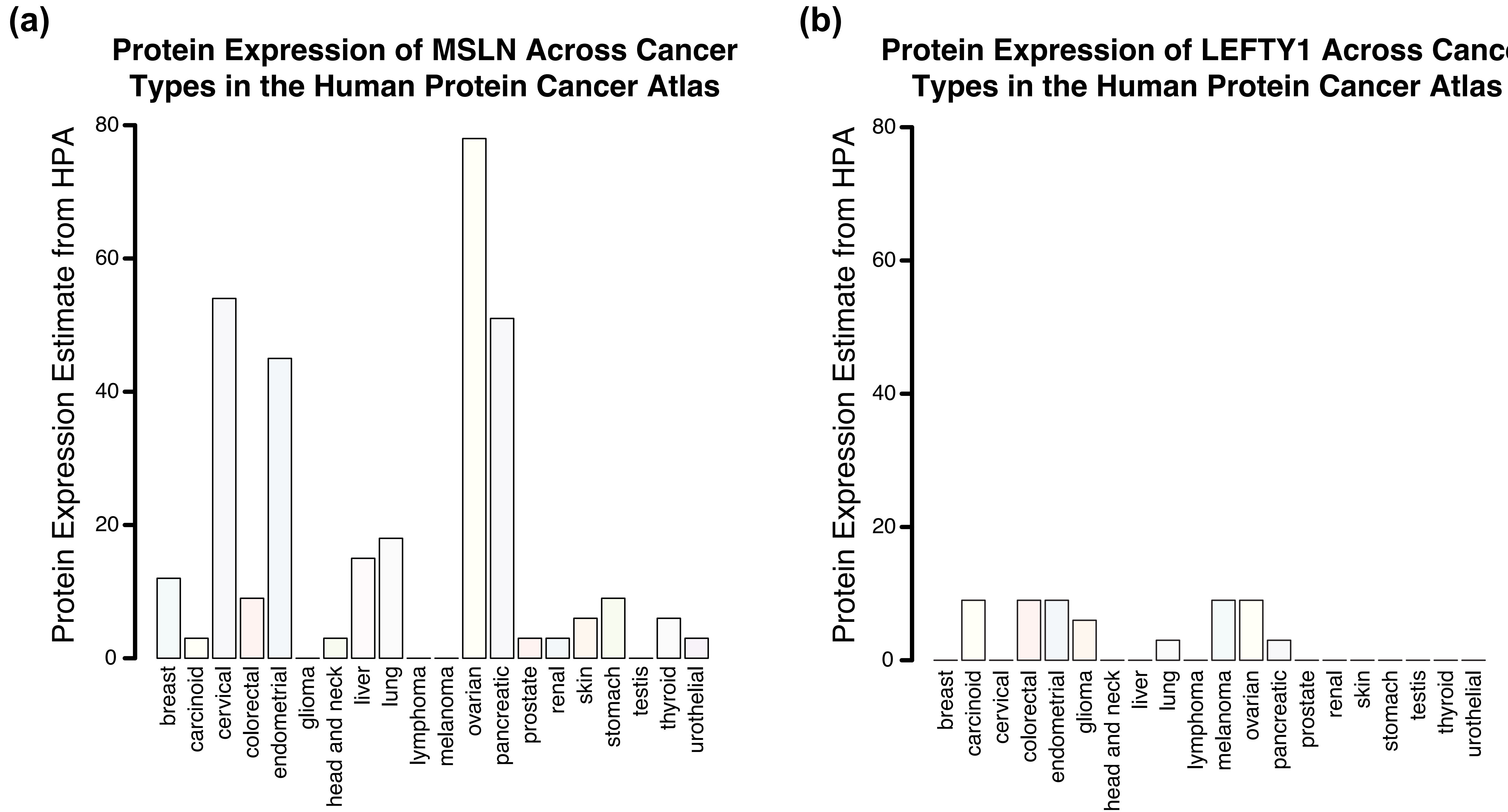

Supplemental Figure 13

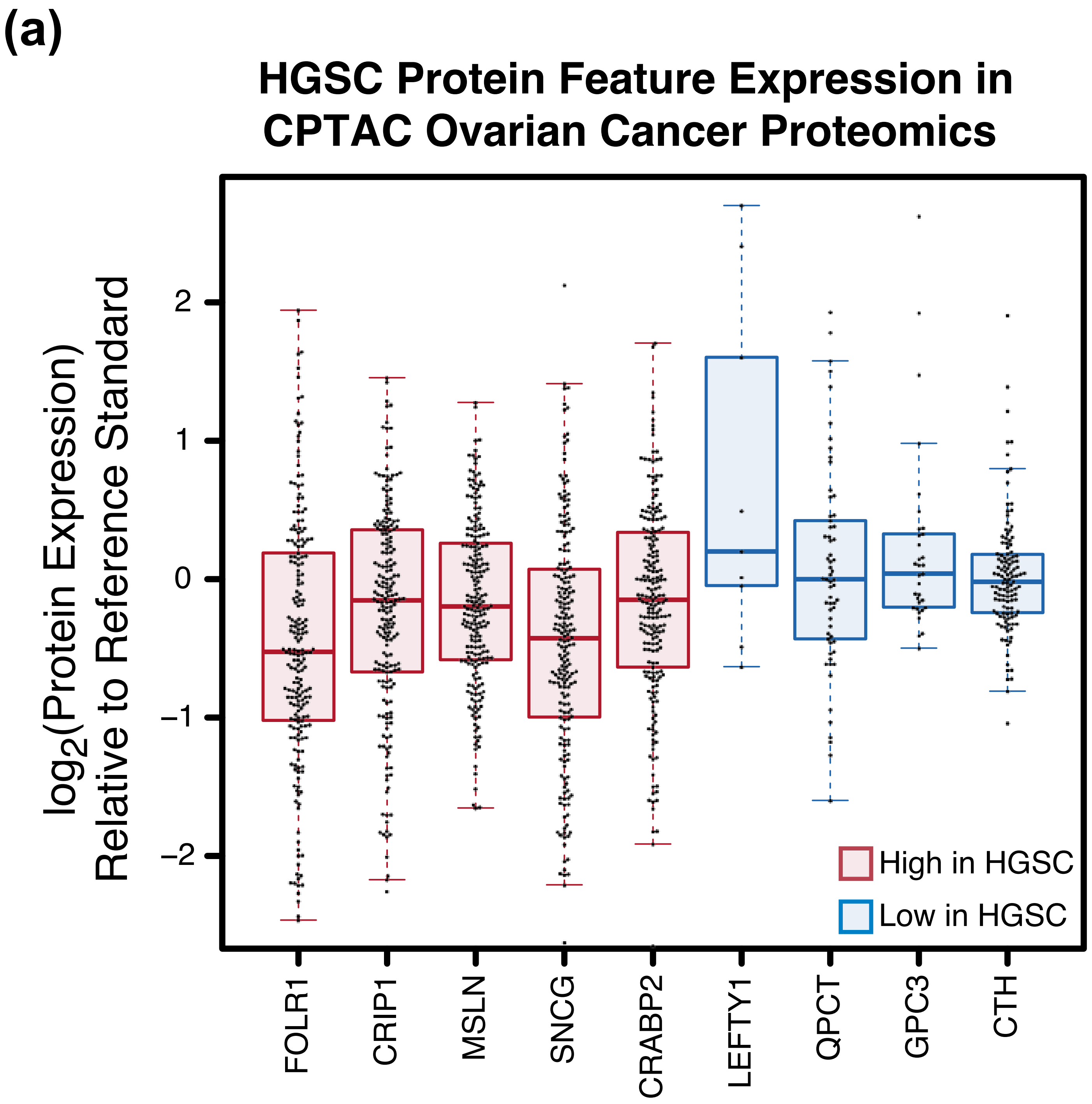

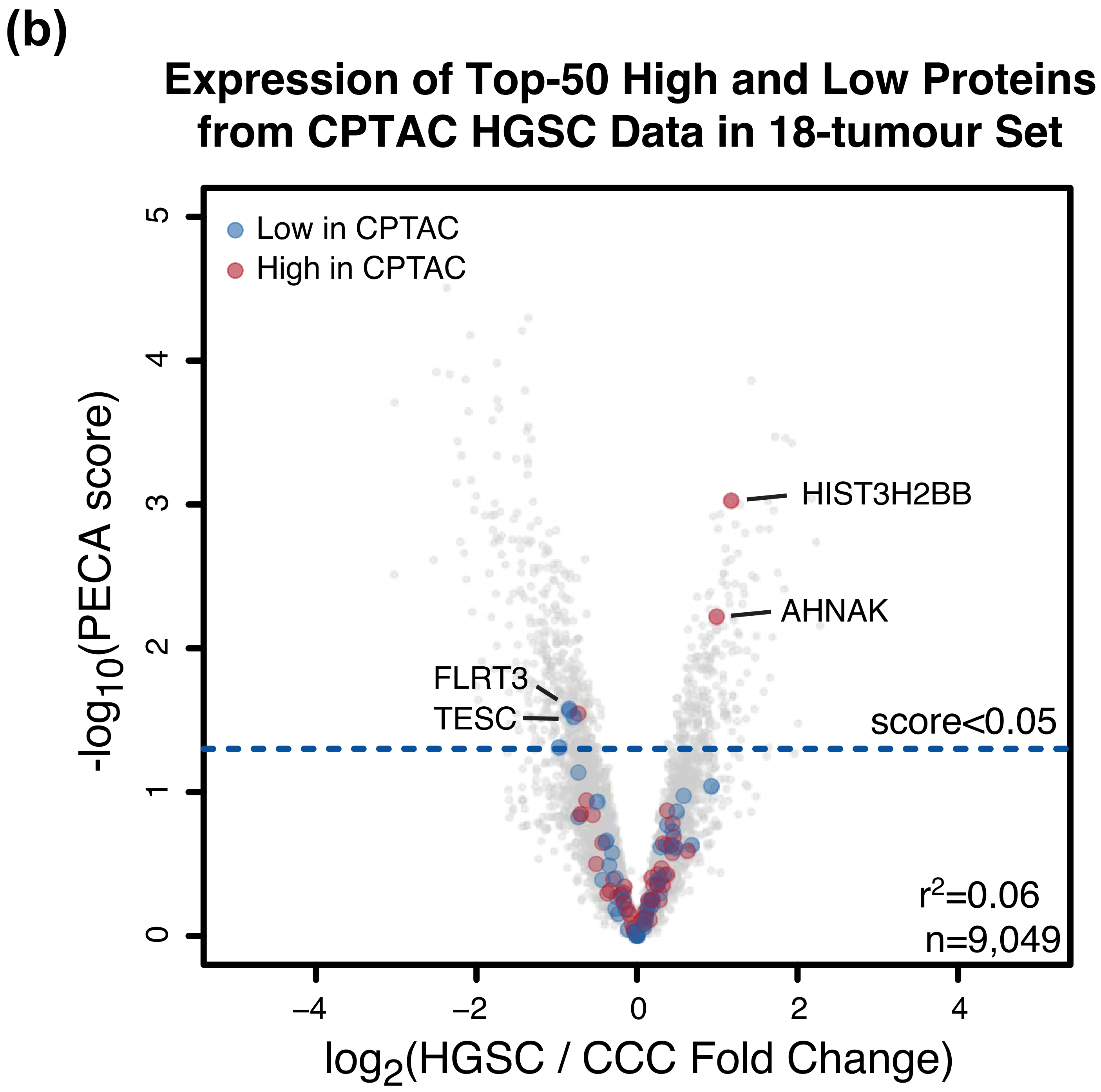

Supplemental Figure 14

(a)

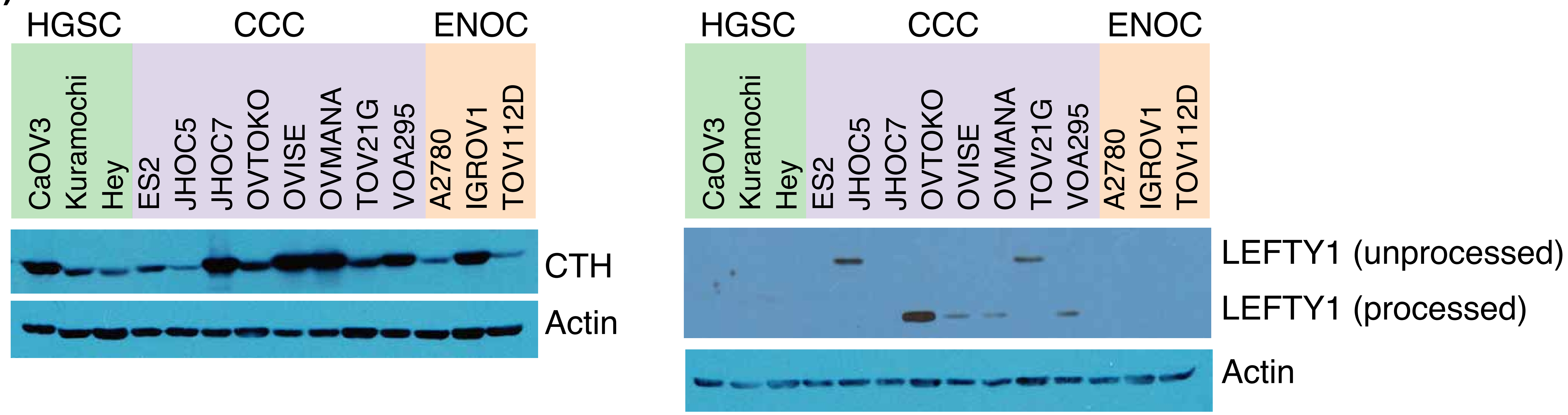

(b)

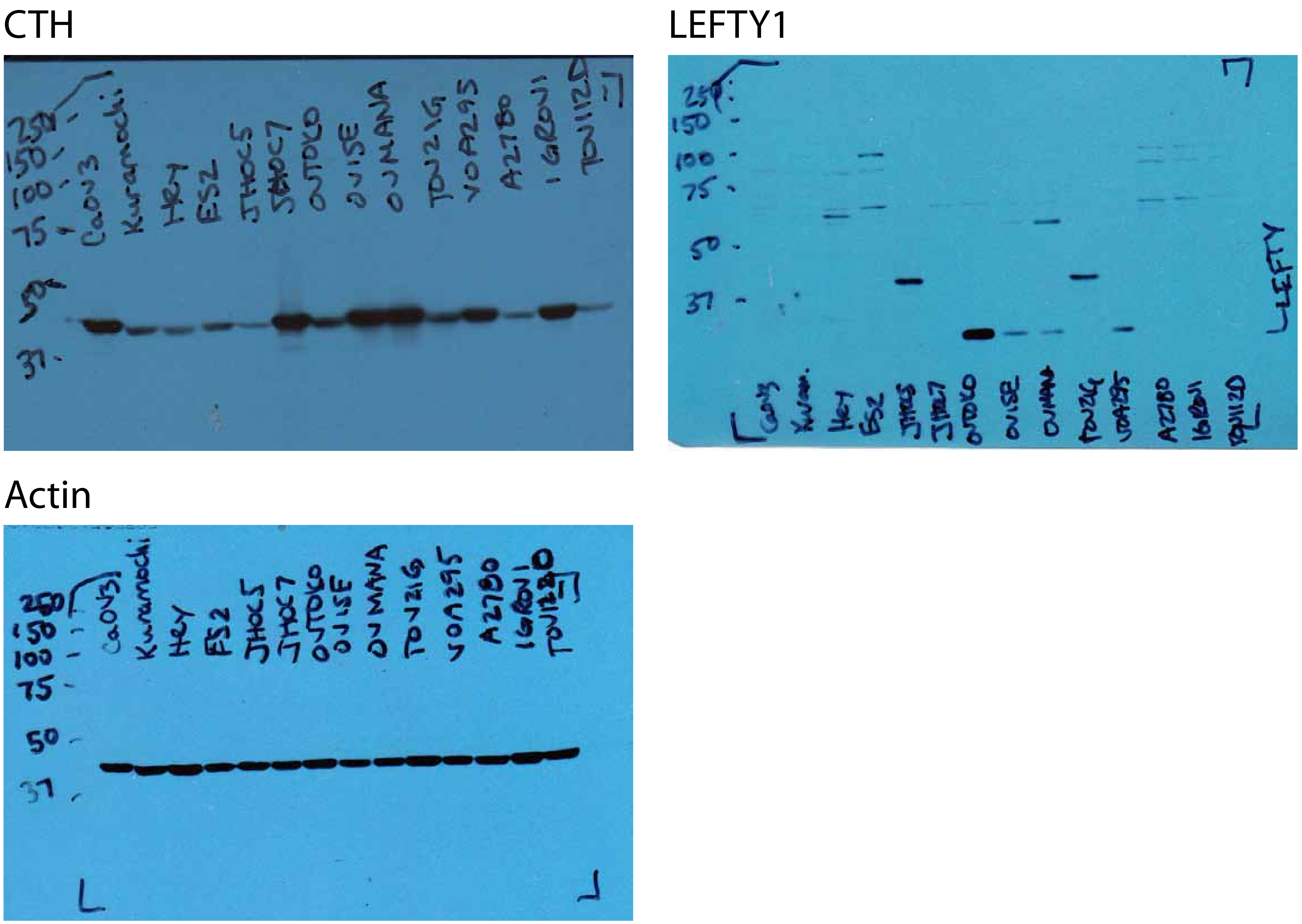

Supplement: Supplementary Information [file srep34949-s1.pdf]
